# Synaptic Adaptations in the Amygdala During Alprazolam Withdrawal: Insights from a Female Rat Model of Benzodiazepine Withdrawal

**DOI:** 10.3390/ph19091433

**Published:** 2026-09-10

**Authors:** Tamara Radukic, Jelena Martinovic, Milorad Dragic, Milica Zeljkovic Jovanovic, Danica Popovic, Nebojsa Jasnic, Andjela Stekic, Marina Zaric Kontic

**Affiliations:** 1 Department of Molecular Biology and Endocrinology, VINČA Institute of Nuclear Sciences—National Institute of the Republic of Serbia, University of Belgrade, Mike Petrovica 12-14, 11351 Belgrade, Serbia; tamara.radukic@vin.bg.ac.rs (T.R.); jelenazlatkovic@vin.bg.ac.rs (J.M.); milorad.dragic@bio.bg.ac.rs (M.D.); 2 Center for Translational Neuroscience, Faculty of Biology, University of Belgrade, Studentski trg 16, 11158 Belgrade, Serbia; milica.zeljkovic@bio.bg.ac.rs (M.Z.J.); danica.popovic@bio.bg.ac.rs (D.P.); andjela.stekic@bio.bg.ac.rs (A.S.); 3 Department for Animal and Human Physiology, Faculty of Biology, University of Belgrade, Studentski trg 16, 11158 Belgrade, Serbia; jasnicn@bio.bg.ac.rs

**Keywords:** alprazolam withdrawal, benzodiazepine withdrawal, amygdala, synaptic plasticity, glutamatergic signaling, GABAergic neurotransmission, dendritic spines, anxiety-like behavior, female rats

## Abstract

**Background/Objectives**: Benzodiazepine (BZD) withdrawal is frequently associated with altered emotional reactivity and behavior. Given the higher prevalence of anxiety disorders and the more frequent use of BZDs among women, understanding the neurobiology of BZD withdrawal in females is particularly important as it remains elusive. **Methods**: Adult female Wistar rats received 8 days of chronic, escalating-dose alprazolam (ALP) treatment followed by spontaneous withdrawal assessed at 36 h and 96 h from the last dose. Behavioral assessment was performed using the Open Field Test. Synaptic protein expression was analyzed by Western blot; neurotransmitter and neuromodulator levels were determined using high-performance liquid chromatography (HPLC) and commercial assay kits; and dendritic spine density and morphology were evaluated by Golgi–Cox staining. **Results**: ALP withdrawal significantly increased levels of anxiety-like behavior while reducing locomotor and exploratory activity. These behavioral changes were accompanied by increased glutamate levels and protein expression of vGluT1, GluR1, NR1, NR2B, and PSD-95, followed by delayed upregulation of EAAT1 and EAAT2. The GABAergic system exhibited heterogeneous remodeling, characterized by increased GAD65, α1-containing GABA_A_ receptors, and gephyrin, and reduced vGAT expression. Dopamine and serotonin levels remained unchanged, whereas D1 receptor levels were transiently reduced during 36 h withdrawal. The discontinuation of ALP was further associated with temporally selective dendritic spine remodeling, characterized by an early, sustained reduction in branched spine density, a transient increase in filopodia at 36 h, and a reduction in total spine density at 96 h. **Conclusions**: These findings demonstrate that ALP withdrawal leads to coordinated neurochemical, molecular, and structural remodeling within the amygdala, involving both excitatory and inhibitory synaptic compartments.

## 1. Introduction

Benzodiazepines (BZDs) are among the most widely prescribed psychoactive medications and are extensively used to treat anxiety and fear-related disorders, insomnia, epilepsy, and other neurological and psychiatric conditions. However, BZDs are also among the most frequently misused prescription drugs and carry a substantial risk of abuse, tolerance, physical dependence, and development of withdrawal symptoms after dose reduction or treatment discontinuation [1,2,3,4]. Benzodiazepine withdrawal syndrome includes a wide range of psychological, behavioral, cognitive, and physical symptoms such as increased anxiety, hyperarousal, irritability, sleep disturbances, cognitive dysfunction, and affective dysregulation, posing a significant clinical challenge that often complicates treatment discontinuation [2,5,6,7]. Compared to other BZDs, alprazolam (ALP) is one of the most frequently used and most misused [8]. Due to its high potency and relatively short elimination half-life, ALP is associated with an increased risk of dependence and withdrawal-related complications compared with several other BZDs [8,9,10]. Consequently, prolonged ALP exposure provides a useful experimental framework for investigating the neurobiological mechanisms underlying BZD withdrawal.

Because the BZD therapeutic effects are mostly mediated through positive allosteric modulation of γ-aminobutyric acid (GABA) type A receptors (GABA_A_Rs), the neurobiology of BZD dependence and withdrawal was primarily interpreted through adaptive changes in GABAergic neurotransmission [1,2,11,12]. Chronic BZD exposure induces multiple compensatory alterations, including changes in GABA_A_R subunit composition, receptor trafficking and coupling, and overall receptor function [2,12]. However, accumulating evidence suggests that withdrawal-associated behavioral and neurobiological abnormalities cannot be fully explained by GABAergic mechanisms alone. Experimental studies have demonstrated that BZD withdrawal is accompanied by increased anxiety-like behavior, enhanced neuronal excitability, and alterations in glutamatergic neurotransmission, supporting the involvement of broader neuroadaptive processes that extend beyond direct GABAergic modulation [1,2,7,10,12,13,14,15].

Among the brain regions involved in emotional regulation and stress responsiveness, the amygdala serves as a key integrative hub that continuously processes sensory, contextual, and interoceptive information, assigns emotional significance to environmental stimuli and shapes behavioral, autonomic, and endocrine responses [16]. Given its central role in coordinating adaptive responses to emotionally salient experiences, the amygdala demonstrates a remarkable capacity for experience-dependent neuroplasticity. Both stress exposure and chronic pharmacological interventions induce structural and molecular remodeling within the amygdala, including changes in neurotransmitter signaling, synaptic organization, and dendritic spine architecture. These adaptations are increasingly recognized as important contributors to persistent changes in emotional reactivity and stress responsiveness [17,18,19,20]. However, despite substantial scientific interest in the neurobiology of BZD withdrawal, the molecular and structural mechanisms underlying withdrawal-induced adaptations within the amygdala remain understudied.

An additional limitation of the available literature is its pronounced sex bias. Although women are more frequently affected by anxiety disorders and receive BZD prescriptions more often than men [21,22], the neurobiology of BZD withdrawal has been studied predominantly in males [14,15]. This discrepancy is especially relevant given the well-documented sex differences in brain structure and function, synaptic plasticity, and stress responsiveness [23,24], all of which may influence neuroadaptive responses to chronic drug exposure and withdrawal [25]. Therefore, the current preclinical findings related to females remain neglected.

In summary, although behavioral manifestations of BZD withdrawal have been documented, much less is known about the accompanying neuroadaptations within the amygdala, especially in females. Therefore, this study used a translationally relevant ALP withdrawal paradigm in adult female Wistar rats and investigated withdrawal-associated alterations across multiple levels of biological organization. Specifically, we examined withdrawal-associated behavioral alterations together with changes in neurotransmitter/neuromodulator content, synaptic protein expression, and dendritic spine morphology within the amygdala. We hypothesized that ALP withdrawal would be associated with coordinated neurochemical and synaptic remodeling within the amygdala.

## 2. Results

### 2.1. Behavioral Alterations Following ALP Withdrawal

Prior to behavioral testing, routine neurological and sensorimotor screening revealed no overt abnormalities in neurological, sensory, vestibular, or gross motor functions in the experimental animals; the corresponding data are available in the Zenodo repository (DOI: 10.5281/zenodo.21197979).

The potential influence of repeated VEH administration on OFT behavior was first evaluated in a separate pilot analysis by comparing baseline measurements with those obtained from the same animals 36 h after the final VEH injection. No significant differences were observed in total distance traveled (*p* = 0.3542, Supplementary Figure S1A), average speed (*p* = 0.3574, Supplementary Figure S1B), number of immobility episodes (*p* = 0.1106, Supplementary Figure S1C), total time immobile (*p* = 0.1462, Supplementary Figure S1D), number of entries into the center (*p* = 0.4881, Supplementary Figure S1E), time spent in the center (*p* = 0.8830, Supplementary Figure S1F), or time spent in the corners of the arena (*p* = 0.4200, Supplementary Figure S1G). Thus, repeated VEH administration did not significantly alter locomotor, exploratory, or OFT parameters related to emotional reactivity relative to baseline.

**Figure 1:**
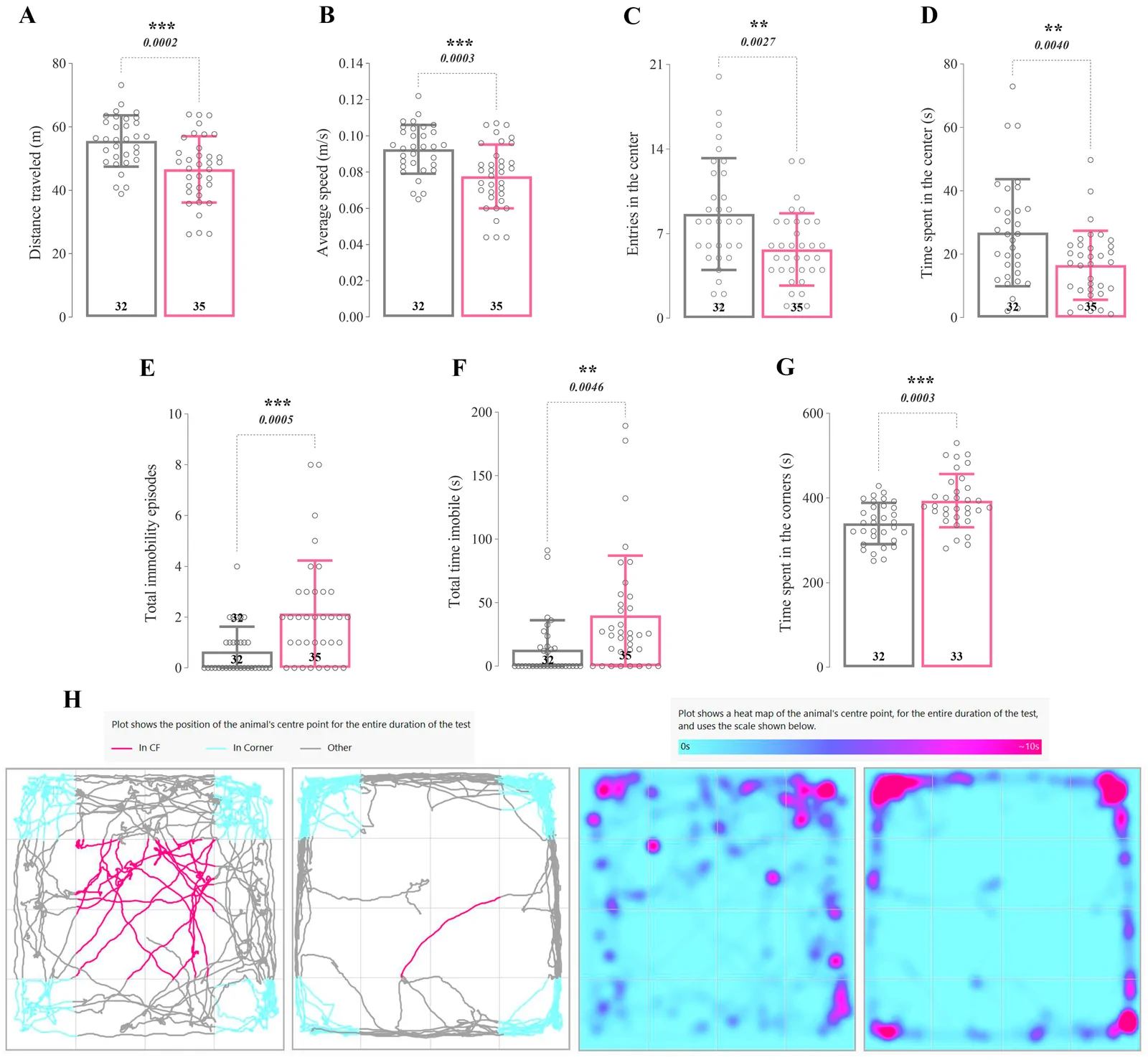
Behavioral parameters assessed in the open field test (OFT) at 36 h of alprazolam withdrawal. The OFT was performed approximately 36 h after the final ALP administration. The following parameters were analyzed: (**A**) total distance traveled, (**B**) average speed, (**C**) number of entries into the central zone, (**D**) time spent in the central zone, (**E**) number of immobility episodes, (**F**) total time spent immobile, and (**G**) time spent in the corners of the arena. (**H**) Representative track plots and heat maps illustrating locomotor and spatial exploration patterns in the VEH and 36 h ALP groups. Dots represent individual animals, and the numbers indicated at the bottom of each graph denote the number of animals included in the corresponding analysis. Data are presented as mean ± SD. Exact *p* values are shown in the graphs, with asterisks indicating statistically significant differences between the VEH and 36 h ALP groups: ** *p* < 0.01, *** *p* < 0.001.

**Figure 2:**
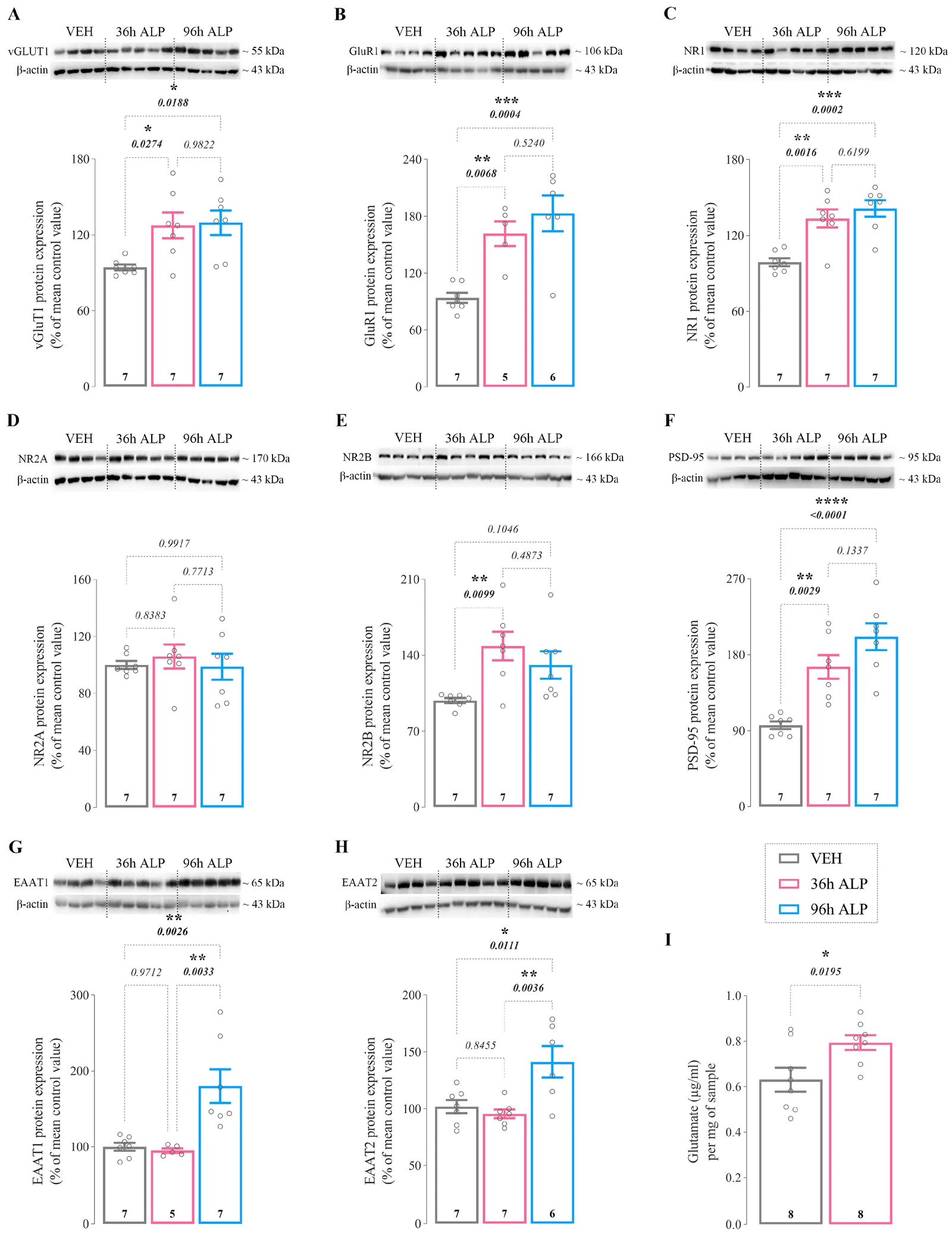
Analysis of components of glutamatergic signaling in synaptosomal fraction of amygdala and glutamate levels. Graphs and representative membranes of (**A**) vGluT1, (**B**) GluR1, (**C**) NR1, (**D**) NR2A, (**E**) NR2B, (**F**) PSD-95, (**G**) EAAT1, (**H**) EAAT2, with their respective β-actin. (**I**) Glutamate levels (µg/mL) per mg of sample. Data are expressed as a percentage of the mean values of the VEH group ± SEM. Dots in the graphs represent the values of individual animals. Numbers at the bottom of the graphs indicate the number of animals included in the analysis. Significance is shown in the graphs as bold, precise numerical values and star symbols indicating significant differences between the VEH and 36 h ALP or 96 h ALP groups: * *p* < 0.05, ** *p* < 0.01, *** *p* < 0.001, **** *p* < 0.0001.

Behavioral assessment in the OFT revealed that 36 h of ALP withdrawal significantly altered both locomotor and parameters related to anxiety-like behaviour (Figure 1). Compared with the VEH group, animals subjected to ALP withdrawal showed a significant reduction in total distance traveled (*t* = 3.902, d_f_ = 65, *p* = 0.0002; Figure 1A) and average speed (*t* = 3.874, d_f_ = 65, *p* = 0.0003; Figure 2B), indicating reduced locomotor and exploratory activity.

Analysis of anxiety-related parameters further demonstrated that ALP withdrawal significantly decreased both the number of entries into the center (*t* = 3.116, d_f_ = 65, *p* = 0.0027; Figure 1C) and time spent in the center of the arena (*t* = 2.982, d_f_ = 65, *p* = 0.0040; Figure 2D). In contrast, animals in the withdrawal group spent significantly more time in the corners of the arena than VEH-treated animals (*t* = 3.847, d_f_ = 63, *p* = 0.0003; Figure 2G).

In addition, ALP withdrawal markedly increased both the number of immobility episodes (*t* = 3.678, d_f_ = 65, *p* = 0.0005; Figure 1E) and the duration of immobility (*t* = 2.933, d_f_ = 65, *p* = 0.0046; Figure 2F). Representative track and heat plots are shown in Figure 2H.

To further distinguish changes in spatial exploration from the concomitant reduction in overall locomotor activity, additional locomotion-adjusted and compartment-specific analyses were performed (Supplementary Figure S2). Distance traveled in the center, expressed as a percentage of the total distance traveled by each animal, was significantly reduced in the 36 h ALP group compared with VEH (*p* = 0.0384; Supplementary Figure S1E). Absolute distance traveled in the center did not differ significantly between groups (*p* = 0.1330; Supplementary Figure S1A), whereas average speed in the center was significantly increased in ALP-withdrawn animals (*p* = 0.0146; Supplementary Figure S1B). In contrast, both distance traveled (*p* = 0.0004; Supplementary Figure S1C) and average speed (*p* = 0.0001; Supplementary Figure S1D) in the periphery were significantly reduced compared with VEH controls. Thus, locomotor alterations during 36 h ALP withdrawal were spatially heterogeneous rather than uniformly expressed across the arena.

### 2.2. Glutamatergic Adaptations in the Amygdaloid Region Following ALP Withdrawal

Glutamatergic adaptations associated with ALP withdrawal were assessed by determining changes in principal glutamatergic signaling components and glutamate levels in the amygdaloid region after 36 h of withdrawal and by analyzing the expression of glutamatergic synaptic proteins at 36 h and 96 h of withdrawal (Figure 2).

Among presynaptic glutamatergic markers, treatment significantly affected the expression of vesicular glutamate transporter 1 (vGluT1; F_(2,18)_ = 5.77, *p* = 0.0115, Figure 2A). Tukey’s post hoc analysis showed significantly higher vGluT1 expression in both the 36 h (*p* = 0.0274) and 96 h (*p* = 0.0188) withdrawal groups compared with VEH, with no significant difference between the two withdrawal time points.

Treatment also significantly affected expression of the GluR1 subunit of α-amino-3-hydroxy-5-methyl-4-isoxazolepropionic acid receptor (AMPAR; F_(2,15)_ = 13.79, *p* = 0.0004, Figure 2B). GluR1 expression was significantly increased after both 36 h (*p* = 0.0068) and 96 h (*p* = 0.0004) of withdrawal compared with the VEH group, without changes between withdrawal time points.

Similarly, treatment significantly affected expression of the N-methyl-D-aspartate receptor (NMDAR) NR1 subunit (F_(2,18)_ = 14.83, *p* = 0.0002, Figure 2C). NR1 expression was increased in both the 36 h (*p* = 0.0016) and 96 h (*p* = 0.0002) withdrawal groups compared to VEH, while no changes were observed between withdrawal time points.

Interestingly, while NR2A subunit expression did not differ among the experimental groups (F_(2,18)_ = 0.27, *p* = 0.7647; Figure 2D), NR2B subunit expression showed significant temporal alterations (F_(2,18)_ = 5.72, *p* = 0.0119, Figure 2E). Post hoc analysis revealed a significant upregulation in the 36 h ALP group compared with VEH (*p* = 0.0099), whereas, interestingly, neither the comparison between VEH and the 96 h ALP group nor between the two withdrawal groups reached statistical significance.

Next, treatment significantly affected expression of the scaffold protein postsynaptic density protein 95 (PSD-95; F_(2,18)_ = 18.09, *p* < 0.0001, Figure 2F). PSD-95 expression was significantly increased in both the 36 h (*p* = 0.029) and 96 h (*p* < 0.0001) ALP groups compared with VEH, with no difference between the two withdrawal time points.

Finally, the glutamate transporters, excitatory amino acid transporter 1 and 2 (EAAT1 and EAAT2, respectively), exhibited a similar pattern of expression following ALP withdrawal, with both proteins being significantly upregulated only after 96 h withdrawal. Treatment significantly affected EAAT1 expression (F_(2,16)_ = 10.88, *p* = 0.0010, Figure 2G). EAAT1 expression was significantly increased in the 96 h ALP group compared with both the VEH (*p* = 0.0026) and 36 h ALP (*p* = 0.0033) groups, whereas no significant difference was observed between the VEH and 36 h ALP groups. Likewise, treatment significantly affected EAAT2 expression (F_(2,17)_ = 8.44, *p* = 0.0028). EAAT2 expression was also significantly increased in the 96 h withdrawal group compared with both the VEH (*p* = 0.0111) and 36 h withdrawal (*p* = 0.0036) groups, whereas no significant difference was observed between the VEH and 36 h withdrawal groups.

In the end, glutamate concentrations were measured in the amygdaloid region and results revealed a significant increase after 36 h of ALP withdrawal (*t* = 2.639, d_f_ = 14, *p* = 0.0195; Figure 2I).

### 2.3. Adaptations in GABAergic Signaling in the Amygdaloid Region Following ALP Withdrawal

**Figure 3:**
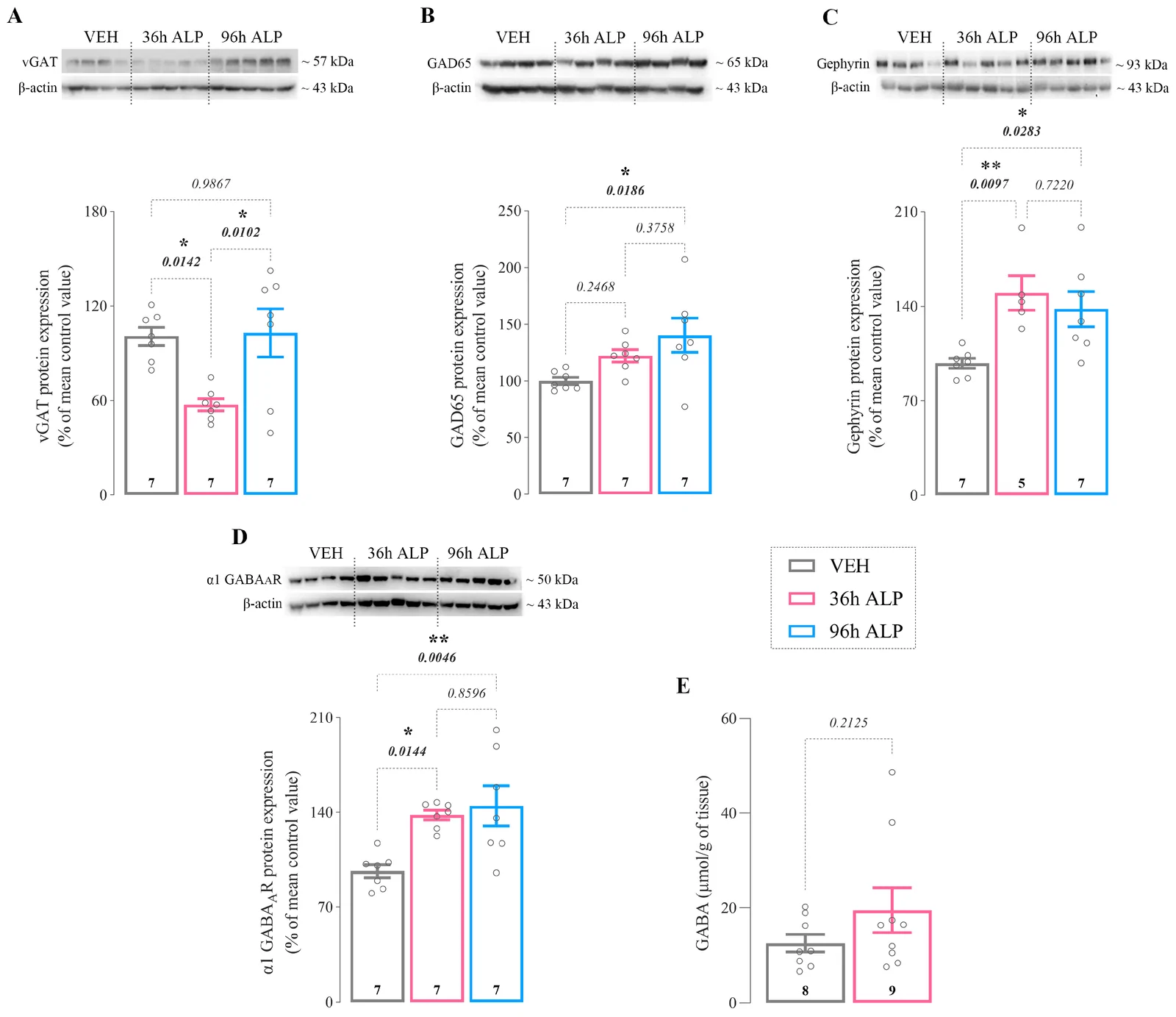
Analysis of components of GABAergic signaling in synaptosomal fraction of amygdala and GABA levels. Graphs and representative membranes of (**A**) vGAT, (**B**) GAD65, (**C**) Gephyrin, (**D**) αGABA_A_R, with their respective β-actin. (**E**) GABA levels (µmol/g of tissue). Data are expressed as a percentage of the mean values of the VEH group ± SEM. Dots in the graphs represent the values of individual animals. Numbers at the bottom of the graphs indicate the number of animals included in the analysis. Significance is shown in the graphs as bold, precise numerical values and star symbols indicating significant differences between the VEH and 36 h ALP or 96 h ALP groups: * *p* < 0.05, ** *p* < 0.01.

GABAergic signaling adaptations associated with ALP withdrawal were assessed by analyzing the expression of key presynaptic and postsynaptic GABAergic proteins at 36 h and 96 h of withdrawal and by measuring GABA levels in the amygdala after 36 h of withdrawal (Figure 3).

Treatment significantly affected expression of the vesicular GABA transporter (vGAT; F_(2,18)_ = 7.01, *p* = 0.0056, Figure 3A). vGAT expression was significantly reduced after 36 h of withdrawal compared with the VEH group (*p* = 0.0142), but returned to control levels after 96 h, resulting in a significant difference between the two withdrawal groups (*p* = 0.0102) but not between the VEH and 96 h groups.

Treatment significantly affected expression of the GABA-synthesizing enzyme glutamate decarboxylase 65 (GAD65; F_(2,18)_ = 4.61, *p* = 0.0241, Figure 3B). GAD65 expression was significantly increased only in the 96 h withdrawal group (*p* = 0.0186) compared with VEH animals, with no significant difference between the two withdrawal time points and between VEH and 36 h timepoint.

Interestingly, treatment significantly affected expression of the inhibitory postsynaptic scaffold protein gephyrin (F_(2,16)_ = 6.87, *p* = 0.0070, Figure 3C). Gephyrin expression was significantly increased after both 36 h (*p* = 0.0097) and 96 h (*p* = 0.0283) of withdrawal compared with the VEH group, with no significant differences between the two withdrawal time points.

Alprazolam withdrawal also significantly affected expression of the α1 subunit of GABA_A_R (F_(2,18)_ = 7.93, *p* = 0.0034, Figure 3D). Expression of the α1 subunit was significantly increased in both the 36 h (*p* = 0.0144) and 96 h withdrawal groups (*p* = 0.0046) compared with VEH, with no significant difference between the withdrawal time points.

Finally, GABA concentration in the amygdala did not differ significantly between the VEH and 36 h ALP groups (*t* = 1.302, d_f_ = 15, *p* = 0.2125; Figure 3E).

### 2.4. Dopaminergic and Serotonergic Adaptations in the Amygdaloid Region Following ALP Withdrawal

**Figure 4:**
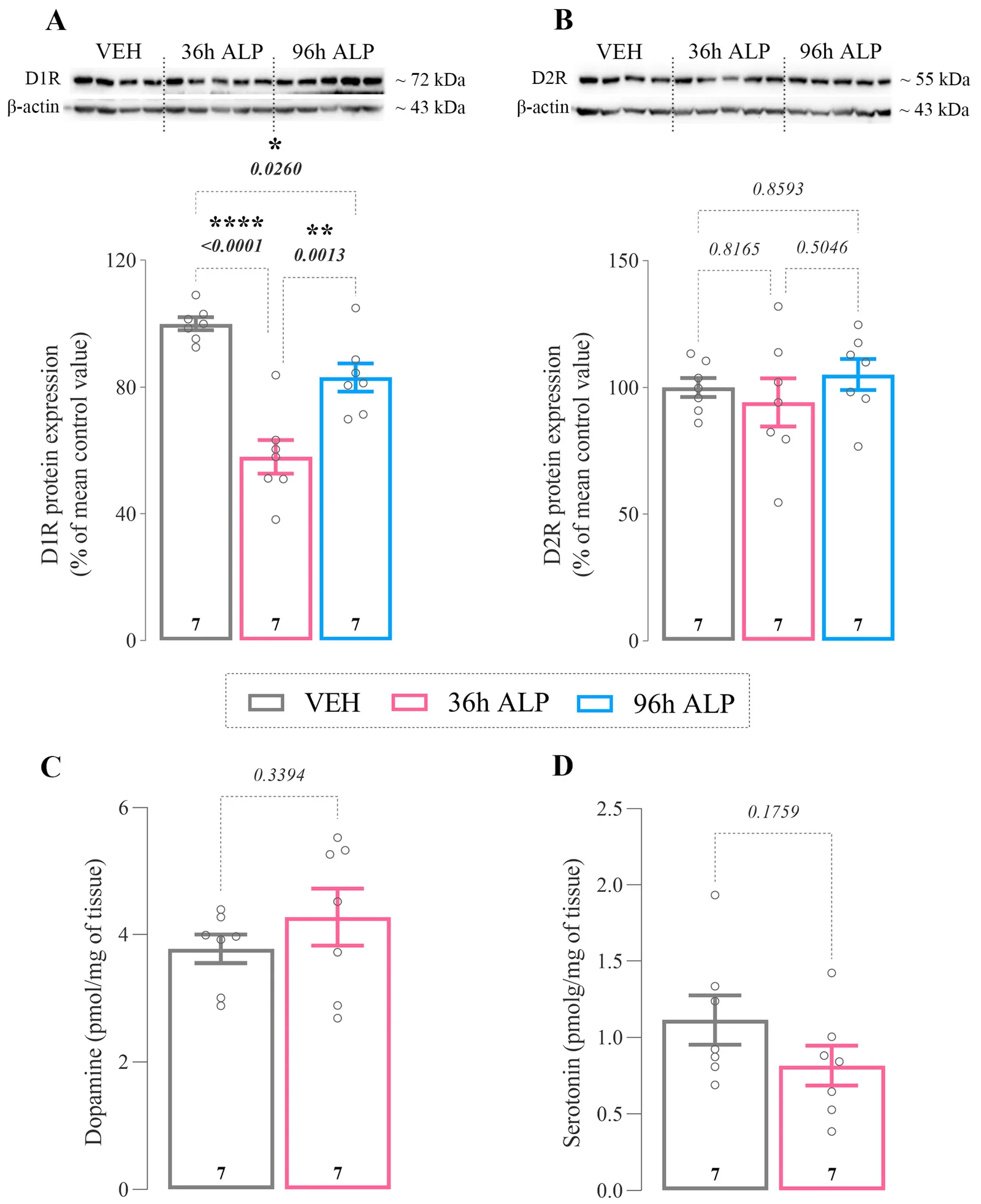
Analysis of dopamine receptors in synaptosomal fraction of amygdala and dopamine and serotonin levels. Graphs and representative membranes of (**A**) D1R, (**B**) D2R, with their respective β-actin. (**C**) Dopamine levels (pmol/mg of tissue), (**D**) Serotonin levels (pmol/mg of tissue). Data are expressed as a percentage of the mean values of the VEH group ± SEM. Dots in the graphs represent the values of individual animals. Numbers at the bottom of the graphs indicate the number of animals included in the analysis. Significance is shown in the graphs as bold, precise numerical values and star symbols indicating significant differences between the VEH and 36 h ALP or 96 h ALP groups: * *p* < 0.05, ** *p* < 0.01, **** *p* < 0.0001.

Dopaminergic adaptations associated with ALP withdrawal were evaluated by measuring the expression of dopamine D1 and D2 receptors (D1R and D2R) at both 36 h and 96 h of withdrawal, as well as dopamine levels in the amygdaloid region after 36 h of withdrawal. Serotonergic adaptations were assessed solely by measuring serotonin levels in the amygdaloid region at the 36 h withdrawal time point (Figure 4).

Treatment significantly affected dopamine D1 receptor (D1R) expression (F_(2,18)_ = 21.01, *p* < 0.0001), whose expression was significantly reduced after 36 h of withdrawal compared with the VEH group (*p* < 0.0001). At 96 h of withdrawal, D1R expression was significantly increased compared with 36 h (*p* = 0.0013) but remained significantly lower than in the VEH group (*p* = 0.0260).

In contrast, dopamine D2 receptor (D2R) expression did not differ among the experimental groups (F_(2,18)_ = 0.64, *p* = 0.5351; Figure 4B). Neither dopamine (*t* = 0.9949, d_f_ = 12, *p* = 0.3394; Figure 4C) nor serotonin (*t* = 1.438, d_f_ = 12, *p* = 0.1759; Figure 4D) concentrations differed significantly between the VEH and 36 h ALP withdrawal groups.

### 2.5. Structural Synaptic Adaptations in the Basolateral Amygdala Following ALP Withdrawal

Structural synaptic adaptations associated with ALP withdrawal were evaluated by analyzing dendritic morphology, dendritic spine density and subtype distribution and synaptophysin expression in the BLA.

**Figure 5:**
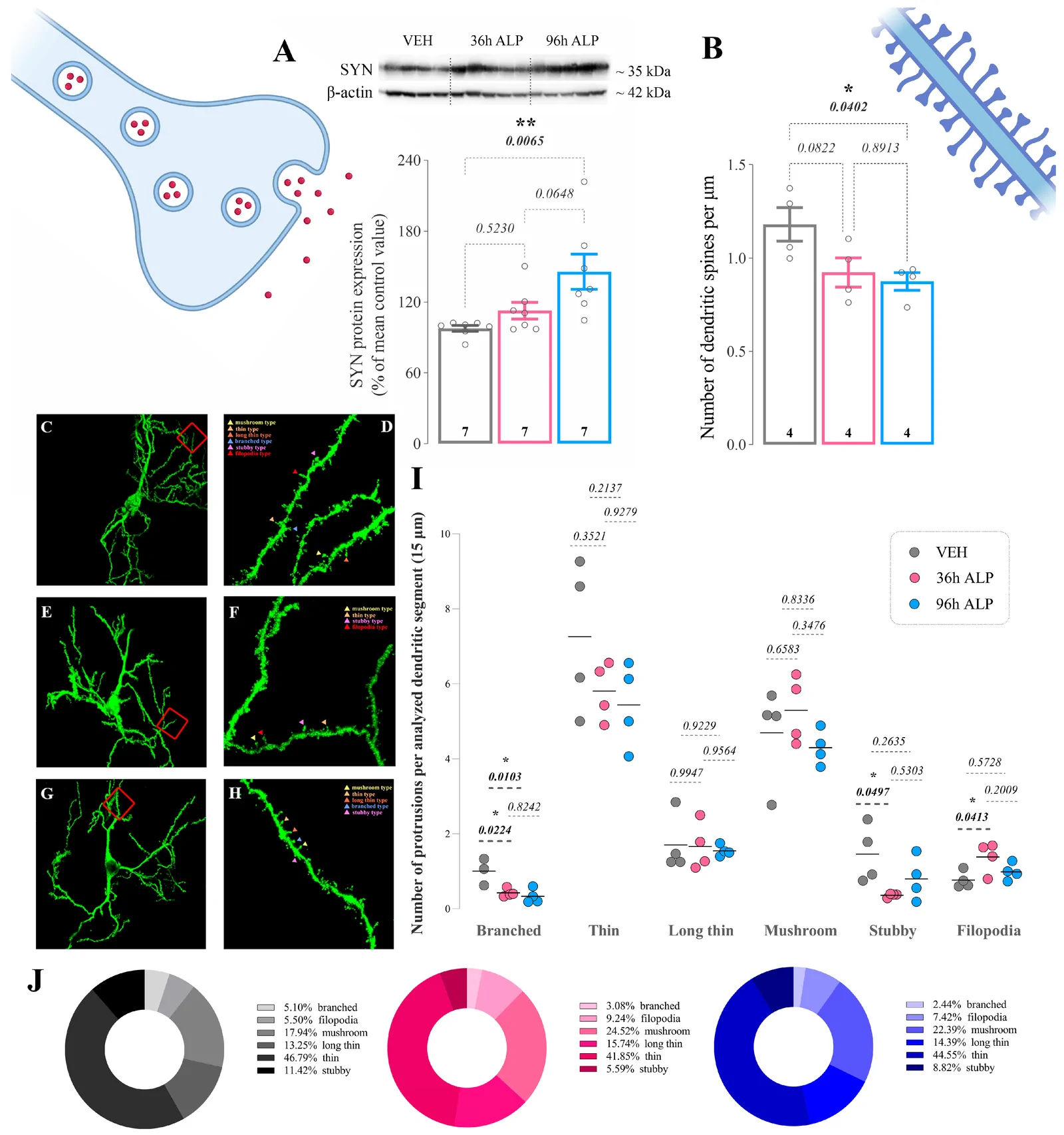
Synaptophysin expression and Golgi–Cox analysis of dendritic spine morphology in pyramidal neurons of the basolateral amygdala during ALP withdrawal. (**A**) Representative Western blot and densitometric analysis of SYN expression in the synaptosomal fraction of the amygdala, with β-actin used as the loading control. SYN expression is presented as a percentage of the mean VEH value ± SEM. (**B**) Total dendritic spine density in BLA pyramidal neurons, expressed as the number of dendritic spines per μm. For dendritic spine analyses, measurements obtained from the analyzed neurons and dendritic segments within each animal were aggregated to generate a single animal-level mean, with individual animals treated as the independent experimental units (*n* = 4 animals per group). (**C**,**E**,**G**) Representative BLA pyramidal neurons from the VEH, 36 h ALP, and 96 h ALP groups, respectively. For all representative neurons, apical dendritic branches are oriented toward the upper part of the image. Red boxes indicate the dendritic regions shown at higher magnification in the corresponding panels (**D**,**F**,**H)**. (**D**,**F**,**H**) Representative dendritic segments from the corresponding experimental groups, illustrating the morphological classification of individual dendritic spine subtypes. Colored triangles indicate mushroom, thin, long-thin, branched, stubby, and filopodia spine types, as specified in the image legends. (**I**) Animal-level analysis of individual dendritic spine subtypes, expressed as the number of protrusions per analyzed 15-μm dendritic segment. (**J**) Relative distribution of dendritic spine subtypes, expressed as the percentage contribution of each subtype to the total spine population in the VEH, 36 h ALP, and 96 h ALP groups, shown from left to right, respectively. Individual dots in the quantitative graphs represent individual animals. Data are presented as mean ± SEM. Exact *p* values are shown in the graphs, with statistically significant comparisons additionally indicated by asterisks: * *p* < 0.05, ** *p* < 0.01, *p* < 0.001. The schematic elements in panels (**A**,**B**) were created in BioRender. Zaric kontic, M. (2026) https://BioRender.com/26i5ggv (accessed on 31 August 2026). BioRender. Zaric Kontic, M. (2026) https://BioRender.com/rzmsb3o.

Synaptophysin (SYN) expression was first evaluated as a marker of presynaptic terminals. Treatment significantly affected synaptophysin expression (F_(2,18)_ = 6.5, *p* = 0.0075, Figure 5A). Synaptophysin expression was significantly increased after 96 h of withdrawal compared with the VEH group (*p* = 0.0065), whereas no significant differences were observed between the VEH and 36 h withdrawal groups or between the two withdrawal time points (Figure 5A).

To exclude differences in dendritic sampling as a potential confounding factor, the lengths of analyzed dendritic segments were first compared and found not to differ among the experimental groups (F_(2,173)_ = 0.50, *p* = 0.6014; data are available in the Zenodo repository; DOI: 10.5281/zenodo.21197979). Post hoc analysis likewise confirmed that no pairwise differences reached statistical significance.

Analysis of total dendritic spine density revealed a significant effect of withdrawal (F_(2,9)_ = 4.972, *p* = 0.0351; Figure 5B). Total spine density showed a progressive decrease throughout the withdrawal, reaching a significant reduction at 96 h compared with the VEH group (*p* = 0.0402), whereas no significant difference was observed at 36 h (Figure 5B).

To identify which dendritic spine populations contributed to the reduction in total spine density of BLA, individual spine subtypes were subsequently analyzed. Branched spine density was significantly decreased (F_(2,8)_ = 8.834, *p* = 0.0094; Figure 5I) in both withdrawal groups relative to VEH (*p* = 0.0224 and *p* = 0.0103, respectively), with no significant difference between the 36 h and 96 h withdrawal groups. Stubby spine density did not show a significant overall effect of withdrawal (F_(2,9)_ = 3.962, *p* = 0.0583, Figure 5I); however, post hoc analysis revealed a significant reduction after 36 h (*p* = 0.0497) compared with the VEH group, while no significant differences were observed between the two withdrawal groups or between the 96 h withdrawal to VEH group. Filopodia spine density showed a significant effect of withdrawal (F_(2,9)_ = 4.364, *p* = 0.0473; Figure 5I), with a significant increase at 36 h compared with the VEH group (*p* = 0.0413), whereas no significant difference was observed between the two withdrawal groups or 96 h compared to the VEH group. In contrast, mushroom (F_(2,9)_ = 1.101, *p* = 0.3734, Figure 5I), thin (F_(2,9)_ = 1.878, *p* = 0.2081, Figure 5I) and long-thin spine densities (F_(2,9)_ = 0.07933, *p* = 0.9244, Figure 5I) did not differ significantly among the experimental groups. Representative BLA pyramidal neurons and corresponding dendritic segments from the VEH, 36 h ALP, and 96 h ALP groups are shown in Figure 5C–H.

## 3. Discussion

The present study provides a comprehensive characterization of ALP withdrawal in adult female rats by integrating behavioral assessment with neurochemical, synaptic, and dendritic spine analyses. Whereas previous experimental studies have largely focused on individual aspects of BZD withdrawal, such as behavioral manifestations or receptor adaptations, the present study examined complementary levels of organization within the same experimental framework. This integrative approach offers a broader understanding of the neurobiological processes underlying BZD withdrawal.

A principal finding of the study is that 36 h ALP withdrawal produced a highly consistent behavioral phenotype in the OFT. Rather than relying on a single behavioral parameter, withdrawal was characterized by reduced locomotor and exploratory activity, decreased center exploration, increased corner occupancy, and prolonged immobility, with all measured parameters indicating the same behavioral outcome. The consistency of these alterations across a relatively large experimental cohort further strengthens the robustness of the observed withdrawal phenotype. These findings are consistent with previous studies demonstrating increased anxiety-like behavior following withdrawal after chronic treatment with different BZDs, despite substantial differences in treatment duration, dosing regimens, and behavioral paradigms [26,27,28,29,30,31]. Nevertheless, the observed reduced locomotor activity during withdrawal requires careful interpretation because it could theoretically reflect residual sedative drug effects rather than withdrawal itself. Importantly, the additional locomotion-adjusted, compartment-specific analyses provide a more nuanced interpretation of the behavioral phenotype observed during early ALP withdrawal. Although overall locomotor activity was reduced, this effect was not uniformly expressed across the OFT arena. Both distance traveled and average speed were markedly reduced in the periphery, whereas absolute distance traveled in the center remained unchanged. Moreover, when distance traveled in the center was normalized to the total distance traveled by each animal, the relative proportion of locomotor activity occurring within the center remained significantly reduced. This finding indicates that the altered pattern of center exploration cannot be attributed solely to the overall reduction in locomotor activity. Interestingly, ALP-withdrawn animals exhibited increased movement speed specifically within the center despite reduced locomotor activity in the periphery. Given the exposed and potentially anxiogenic nature of the OFT center, this spatially selective increase in speed may reflect more rapid traversal of the center rather than sustained exploration of this area. Taken together with the reduced proportion of total locomotion occurring within the center, these findings suggest an altered spatial organization of exploratory behavior during early withdrawal rather than simple generalized locomotor suppression. Nevertheless, because movement speed alone does not directly measure escape or avoidance motivation, this interpretation should be considered cautiously. Additional preliminary, unpublished analyses performed during revision further argue against this possibility. Using a validated gas chromatography-mass spectrometry method, parent ALP was not detected in hippocampal tissue at either 36 h or 96 h after the final administration (<LOD), whereas ALP was readily detected in brain tissue shortly after administration in positive-control animals. Although these preliminary measurements were performed in the hippocampus because residual amygdala tissue was unavailable for analysis, they provide additional evidence against substantial residual cerebral ALP exposure at the withdrawal time points examined. This interpretation is further supported by our previous findings obtained during active ALP exposure. In our previous study, rats receiving prolonged ALP treatment (2 mg/kg/day for 14 days) were tested shortly after the final drug administration, when ALP was still pharmacologically active. Despite ongoing drug exposure, speed and distance travelled in the OFT did not differ from VEH animals, indicating the possible development of tolerance to the sedative effects of ALP [10]. Taken together, the absence of detectable parent ALP at the withdrawal endpoints in our preliminary analysis and the lack of locomotor suppression observed previously during active ALP exposure argue against residual ALP-mediated sedation as the primary explanation for the reduced locomotor activity observed at 36 h of withdrawal. Importantly, reduced locomotor activity should not be interpreted as a nonspecific motor deficit. Considered together with decreased center exploration, increased corner occupancy, and prolonged immobility, it forms part of a coherent behavioral profile consistent with increased anxiety-like behavior and reduced exploratory drive. Given the central role of the amygdala in animal emotional reactivity and defensive behavioral responses [16,32], these behavioral findings provide a relevant context for considering the neurochemical, molecular, and structural adaptations independently identified during withdrawal.

In separate experimental cohorts, ALP withdrawal was associated with a distinct pattern of molecular adaptations in the amygdala, with glutamatergic changes forming the most coherent biological profile. Among these adaptations, glutamatergic changes formed the most coherent biological pattern. Withdrawal was associated with increased glutamate content and elevated expression of vGluT1, GluR1, NR1, NR2B, PSD95, and synaptophysin, suggesting that multiple components of excitatory synapses are affected during ALP withdrawal. These findings align with accumulating evidence that enhanced glutamatergic signaling is one of the major neurobiological features of BZD withdrawal [5,13,33]. Pharmacological studies have repeatedly shown that attenuation of glutamatergic transmission, particularly through NMDAR antagonism, alleviates withdrawal manifestations, supporting a functional contribution of glutamate-dependent mechanisms to withdrawal-associated neuronal hyperexcitability [13,30,34,35,36,37,38,39]. Together, these studies suggest that excessive glutamatergic activity is not merely associated with withdrawal but may actively contribute to its behavioral and neurobiological manifestations. The present findings are also consistent with previous observations by Das and colleagues (2008), who demonstrated that BZD withdrawal enhances AMPAR-mediated synaptic function and promotes synaptic incorporation of GluA1-containing AMPAR in the hippocampus. These results suggested that BZD withdrawal engages mechanisms resembling activity-dependent synaptic plasticity rather than simply representing a consequence of reduced GABAergic inhibition [39]. The present study extends these observations to the amygdala by demonstrating that withdrawal is accompanied by coordinated glutamatergic adaptations involving presynaptic, postsynaptic, and synaptic scaffolding components within a clinically relevant ALP withdrawal paradigm in female rats. Collectively, these findings suggest that glutamatergic adaptations during ALP withdrawal are not restricted to individual receptors or neurotransmitter availability but involve multiple molecular components that regulate excitatory synaptic transmission.

Interestingly, glutamatergic adaptations during ALP withdrawal were not restricted to neuronal components of excitatory synapses. At the later 96 h withdrawal time point, both EAAT1 and EAAT2 were significantly upregulated. These high-affinity glutamate transporters are responsible for most extracellular glutamate clearance and play a critical role in maintaining glutamate homeostasis by limiting spillover and excessive activation of glutamate receptors. EAAT1 is expressed predominantly by astrocytes, whereas EAAT2, although primarily localized to perisynaptic astrocytic processes, has also been detected at a subset of presynaptic glutamatergic terminals [40,41,42]. Because Western blot analyses were performed using synaptosomal fractions, which are enriched in synaptic elements but may retain perisynaptic astrocytic processes that remain structurally associated with synaptic terminals, the precise cellular origin of the observed increase cannot be unequivocally determined. Therefore, the present findings should be interpreted with caution. Nevertheless, the increased protein expression of EAAT1 and EAAT2 at 96 h is consistent with the activation of homeostatic mechanisms at the neuron–astrocyte interface that may enhance glutamate clearance in response to the accompanying glutamatergic adaptations during withdrawal. Whether these changes predominantly reflect astrocytic, neuronal, or coordinated tripartite synaptic adaptations remains to be established using cell type-specific approaches.

In contrast to the coordinated glutamatergic alterations observed during ALP withdrawal, molecular changes within the GABAergic system followed a considerably more heterogeneous pattern. Tissue GABA content tended to increase, although this change did not reach statistical significance, while proteins involved in GABA synthesis, vesicular transport, receptor composition, and postsynaptic organization showed divergent patterns of regulation. This observation suggests that withdrawal affects multiple levels of inhibitory synaptic function rather than simply reducing GABAergic activity. Previous experimental studies likewise demonstrate that chronic BZD exposure and subsequent withdrawal represent distinct neurobiological states characterized by dynamic, region-specific GABAergic adaptations [2,33]. Using female rats chronically treated with triazolam for four weeks, Ramsey-Williams and Carter reported that the molecular profile observed after 24 h of withdrawal differed markedly from that present during chronic drug exposure, indicating that withdrawal engages a distinct program of receptor regulation rather than merely extending adaptations induced by prolonged BZD treatment [43]. Moreover, these changes varied considerably among brain regions and GABA_A_R subunits, emphasizing that GABAergic adaptations during withdrawal are highly context and region dependent [43]. Similarly, Follesa and colleagues showed that withdrawal following chronic diazepam exposure induced selective remodeling of GABA_A_R subunit composition in cerebellar granule cell cultures. Whereas chronic diazepam treatment reduced α1 and γ2 subunit expression, 6 h of withdrawal produced a marked increase in α4 expression together with a further reduction in α1, indicating that receptor remodeling is specifically associated with the withdrawal state rather than chronic drug exposure itself [44]. Collectively, these studies suggest that BZD withdrawal is characterized by active modification of inhibitory synapses, although the direction of individual molecular changes depends on the experimental model, withdrawal interval, brain region, and receptor subtype and subunit examined. Within this context, the divergent regulation of GABAergic markers observed in our study provides additional insight into the mechanisms through which inhibitory synapses respond to ALP withdrawal. Increased GAD65 expression suggests an enhanced capacity for activity-dependent GABA synthesis at presynaptic terminals. Because GAD65 preferentially supplies GABA required for synaptic neurotransmission rather than maintaining basal intracellular GABA pools, its upregulation may reflect an increased demand for transmitter synthesis during periods of elevated neuronal activity. In line with this interpretation, tissue GABA levels exhibited a consistent increasing tendency, indicating that alterations in synaptic protein expression may precede detectable changes in total tissue neurotransmitter content. Interestingly, increased GAD65 expression was accompanied by reduced vGAT abundance, suggesting that enhanced GABA synthesis is not necessarily paralleled by increased vesicular loading of the neurotransmitter. Since vGAT is essential for packaging GABA into synaptic vesicles before release [45,46], these findings suggest that distinct presynaptic components of inhibitory neurotransmission may be regulated independently during withdrawal. Such dissociation between GABA synthesis and vesicular loading may represent a homeostatic mechanism that restrains excessive GABA release despite enhanced GABA production. The postsynaptic compartment showed yet another pattern of adaptation. Gephyrin, the principal scaffold protein responsible for clustering and stabilizing synaptic GABA_A_R [46,47], was increased together with the α1 receptor subunit. Although these findings cannot be taken as evidence of enhanced inhibitory function per se, they indicate that withdrawal is accompanied by modifications of inhibitory postsynaptic architecture. Increased expression of both proteins is therefore consistent with structural adjustments at inhibitory synapses, potentially reflecting an adaptive response to the accompanying remodeling of excitatory synaptic components. Taken together, these observations indicate that ALP withdrawal does not affect inhibitory synapses through a single molecular mechanism. Instead, distinct regulatory patterns across GABAergic synaptic proteins suggest coordinated remodeling of inhibitory synapses during withdrawal. Whether these molecular changes ultimately alter inhibitory efficacy or functionally modify the balance between excitatory and inhibitory transmission cannot be determined from the present data and will require future electrophysiological studies. In addition to glutamatergic and GABAergic adaptations, ALP withdrawal also involved more subtle neuromodulatory changes. Dopamine and serotonin levels showed only nonsignificant tendencies toward increase and decrease, respectively, whereas D1R expression was markedly reduced and D2R expression remained unchanged. These findings suggest a potentially selective alteration of D1R-mediated modulation rather than a generalized disruption of dopaminergic signaling. Given the established role of D1R in regulating glutamatergic transmission, NMDAR/AMPAR function, and synaptic plasticity, reduced D1R expression may influence the integration of excitatory inputs within amygdala circuits [48,49]. Although serotonin levels were not significantly altered, previous studies indicate that serotonergic mechanisms contribute to BZD withdrawal-associated anxiety in a region-specific manner and modulate amygdala-dependent emotional processing [26,27,28]. Together, these findings suggest that neuromodulatory systems may shape the synaptic environment in which withdrawal-associated plasticity develops and provide an additional link between molecular adaptations and the structural remodeling of dendritic spines demonstrated in the study.

The synaptic neuroadaptations observed during ALP withdrawal were accompanied by temporally and morphologically selective remodeling of dendritic spines in BLA pyramidal neurons, providing structural evidence that withdrawal alters the organization of excitatory synapses within the amygdala. Dendritic spines constitute the principal postsynaptic sites of excitatory glutamatergic transmission, and their morphology and turnover are closely associated with synaptic strength, stability, and activity-dependent plasticity [50,51,52]. Importantly, the present animal-level analysis does not indicate a uniform loss of dendritic protrusions. Instead, total spine density showed a progressive decline across withdrawal and became significantly reduced at 96 h, whereas individual spine populations displayed distinct temporal patterns.

Among the analyzed spine subtypes, branched spines exhibited the most consistent alteration, with their density significantly reduced at both 36 h and 96 h of withdrawal. Branched spines represent morphologically complex dendritic protrusions and may form multiple synaptic contacts, suggesting a potentially greater integrative capacity than simpler spine configurations [53]. Their early and sustained reduction may therefore reflect selective remodeling of structurally complex excitatory postsynaptic elements during ALP withdrawal. Notably, this alteration was already evident at 36 h, before a significant reduction in total spine density became detectable, suggesting that remodeling of specific spine populations may precede the later overall decrease in dendritic spine density.

A distinct temporal pattern was observed for filopodia, which were significantly increased at 36 h but did not differ from VEH levels at 96 h. Dendritic filopodia are highly dynamic protrusions involved in structural exploration of the local synaptic environment and can participate in the formation of new synaptic contacts and subsequent spine development [50,51,52]. Their transient increase during early withdrawal may therefore indicate an active phase of structural reorganization in response to the altered synaptic environment. However, because the present analysis provides static morphological measurements rather than longitudinal tracking of individual protrusions, this finding should not be interpreted as direct evidence of de novo synaptogenesis. The normalization of filopodia density at 96 h, coinciding with a significant reduction in total spine density, further suggests that the early increase represents a transient component of structural remodeling rather than a sustained expansion of the dendritic spine population.

Stubby spines showed a less robust pattern. Although pairwise analysis indicated a reduction at 36 h compared with VEH, the overall ANOVA did not reach statistical significance. Given the borderline omnibus effect and the relatively small number of animals included in the Golgi–Cox analysis, this finding should be interpreted cautiously and considered supportive, rather than definitive, evidence of subtype-specific remodeling. In contrast, mushroom, thin, and long-thin spine densities did not differ significantly among experimental groups. The preservation of mushroom spines is particularly noteworthy because these large-headed spines are generally associated with comparatively mature and stable excitatory synaptic contacts [50,51,52]. Thus, the reduction in total spine density observed at 96 h does not appear to reflect generalized disruption of the entire dendritic spine population but rather selective remodeling of particular structural components.

The temporal relationship between these morphological changes and synaptophysin expression provides additional evidence that pre- and postsynaptic compartments may undergo partially dissociable adaptations during withdrawal. At 96 h, when total dendritic spine density was significantly reduced, SYN expression was increased, while several postsynaptic glutamatergic proteins, including GluR1, NR1, and PSD-95, remained elevated. Increased SYN expression should not be interpreted as direct evidence of a greater number of functional synapses, because SYN reflects presynaptic vesicular machinery, whereas Golgi–Cox analysis quantifies postsynaptic dendritic protrusions. Instead, the coexistence of increased SYN expression with reduced dendritic spine density suggests nonparallel remodeling of pre- and postsynaptic synaptic compartments. Increased SYN may reflect altered organization of presynaptic vesicle machinery or compensatory remodeling of remaining synaptic terminals, while the persistent elevation of postsynaptic glutamatergic proteins may indicate molecular reorganization of the remaining excitatory synaptic structures. Whether these adaptations reflect changes in synapse number, synaptic efficacy, or presynaptic release properties cannot be determined from the present measurements and will require direct ultrastructural and functional analyses.

**Figure 6:**
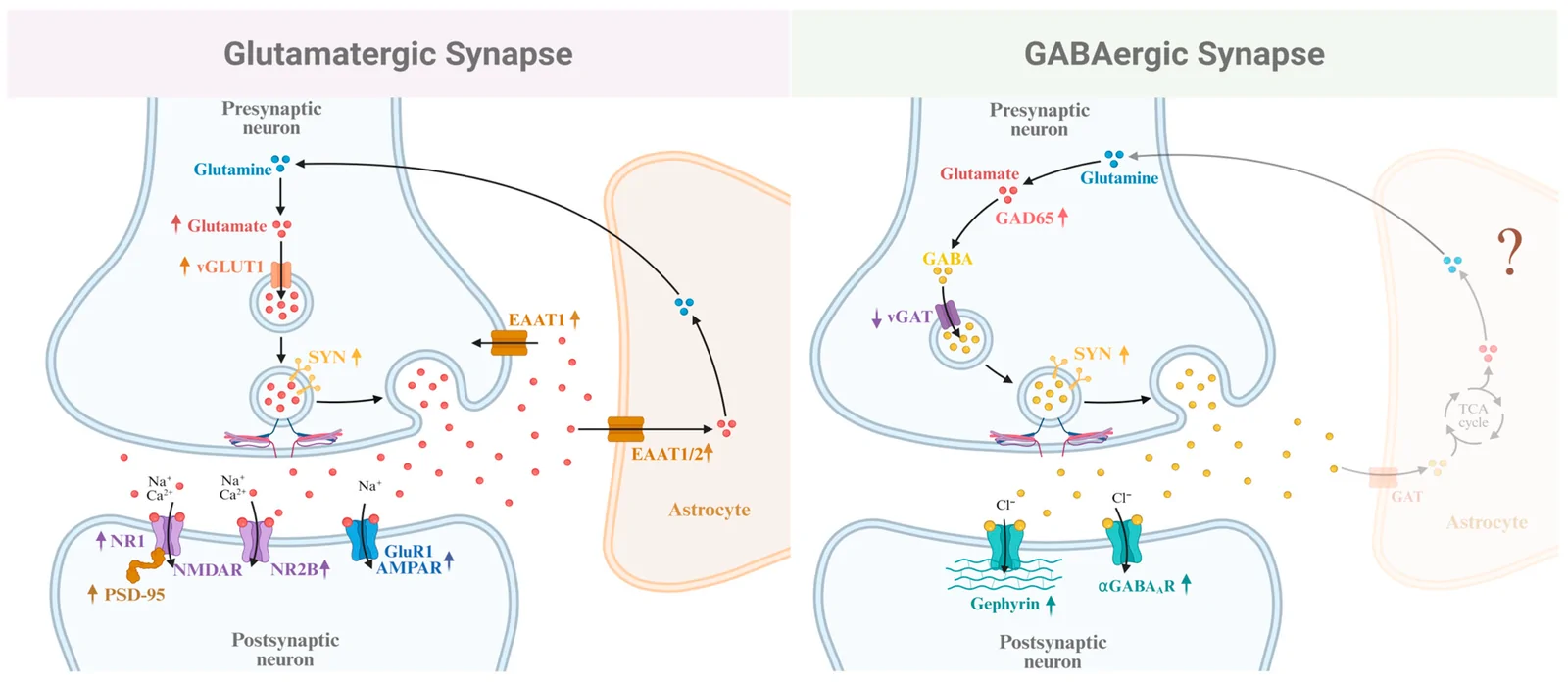
Integrated schematic representation of glutamatergic and GABAergic synaptic adaptations in the amygdala during alprazolam withdrawal. The schematic provides a consolidated representation of the significant neurochemical and molecular alterations observed across the two examined withdrawal time points (36 h and 96 h). Changes detected at either or both withdrawal time points are integrated into a single model to provide an overall view of withdrawal-associated synaptic remodeling rather than separate time-point-specific representations; accordingly, the schematic does not imply that all depicted alterations occur simultaneously or are present at both 36 h and 96 h. Upward (↑) and downward (↓) arrows indicate significant increases and decreases, respectively, relative to the vehicle (VEH) group at one or both withdrawal time points. At the glutamatergic synapse, the integrated changes include increased glutamate levels and increased expression of vGluT1, synaptophysin (SYN), NR1, NR2B, PSD-95, GluR1/AMPAR, EAAT1, and EAAT2. At the GABAergic synapse, the integrated changes include increased expression of GAD65, SYN, gephyrin, and α1-containing GABA_A_R, together with decreased vGAT expression. The neuron–astrocyte glutamine–glutamate cycle is shown schematically to provide metabolic context. The question mark denotes an aspect of astrocyte-associated GABA handling and metabolism that was not directly investigated in the present study and therefore remains unresolved. The schematic summarizes experimentally observed molecular and neurochemical adaptations and does not imply that their functional consequences were directly demonstrated. Created in BioRender. Zaric Kontic, M. (2026) https://BioRender.com/07qc6x3 (accessed on 31 August 2026).

Taken together, these findings suggest that structural remodeling during ALP withdrawal is a dynamic, temporally organized process rather than a generalized loss of excitatory synapses. Early withdrawal was characterized by a sustained reduction in branched spines and a transient increase in filopodia, consistent with active restructuring of the dendritic spine population. By 96 h, this remodeling was accompanied by a significant reduction in overall spine density, while mushroom spines remained preserved and SYN expression increased. Considered together with the persistent increases in GluR1, NR1, and PSD-95 expression, these observations suggest coordinated but temporally distinct remodeling of pre- and postsynaptic components within the BLA during ALP withdrawal. Such structural reorganization may represent part of a broader synaptic adaptation to withdrawal-associated changes in excitatory/inhibitory signaling; however, its functional consequences remain to be established. An integrated schematic summary of the molecular and neurochemical adaptations observed across the two withdrawal time points is presented in Figure 6.

### Study Limitations

This study has several limitations that should be considered when interpreting the findings.

*Lack of functional studies.* Although the observed molecular and structural alterations strongly suggest changes in synaptic organization, no electrophysiological recordings were performed. Consequently, it remains to be *determined* whether the identified neurochemical, molecular, and structural adaptations translate into functional alterations in excitatory and inhibitory synaptic transmission.

*Analysis of dendritic spine morphology.* Whereas multiple neurons and dendritic segments were analyzed within each animal, the number of independent biological replicates was relatively small (n = 4 animals per group). To avoid pseudoreplication, measurements obtained from individual neurons and dendritic segments were aggregated at the animal level, and each animal was treated as an independent experimental unit for statistical analysis. Nevertheless, given the limited number of animals, the spine morphology findings should be considered preliminary and interpreted with appropriate caution, particularly regarding individual spine subtypes. In addition, dendritic spine morphology was assessed at two discrete post-withdrawal time points (36 h and 96 h), providing cross-sectional snapshots rather than longitudinal tracking of structural changes. Therefore, the observed differences cannot be attributed exclusively to the withdrawal period, as they may reflect structural adaptations that developed during prolonged ALP exposure, during withdrawal, or through the combined effects of both phases. Given the dynamic nature of dendritic spines, our analysis does not allow us to determine precisely when these changes arose or how individual spines were remodeled over time. Future studies combining larger independent cohorts with longitudinal in vivo imaging and behavioral assessment will be required to confirm these structural findings and to characterize the dynamics of spine remodeling during BZD withdrawal.

*The control group design.* While the VEH group was assessed 36 h after the final vehicle administration and served as the time-matched control for the 36 h withdrawal group, an independent VEH group examined 96 h after the final vehicle administration was not included. Consequently, although the 96 h findings characterize molecular and structural alterations present at the later withdrawal time point, potential time-dependent changes unrelated to ALP withdrawal cannot be completely excluded. The 96 h findings should therefore be interpreted with this limitation in mind.

*Research using one sex.* This study was conducted exclusively in adult female rats. Therefore, the findings should not be directly extrapolated to males, and future studies will be required to determine whether similar withdrawal-associated adaptations occur in both sexes. The exclusive use of female animals was intentional, given the higher prevalence of anxiety disorders and the more frequent clinical use of BZDs in women. It should be highlighted that the estrous cycle was not monitored in the present study and therefore cycle stage was not included as an experimental variable. Importantly, longitudinal cycle monitoring would require repeated handling and vaginal sampling across the treatment and withdrawal periods, which could itself influence stress-sensitive behavioral and molecular endpoints. Systematic evaluation of estrous cycle–dependent effects would therefore require a dedicated experimental design and should be addressed in future studies.

*Amygdala dissection.* Although amygdala dissections were performed according to stereotaxic coordinates defined in the Paxinos and Watson rat brain atlas [54], precise isolation of the amygdala remains technically challenging because of its anatomical complexity and close proximity to neighboring brain regions. Every effort was made to ensure consistent and anatomically accurate tissue collection across all samples; however, a limited contribution from adjacent tissue cannot be completely excluded.

## 4. Materials and Methods

### 4.1. Animals and Ethical Considerations

All experimental procedures were approved by the Ethics Committee for the Use of Laboratory Animals of Vinca Institute (04/2025, 29 July 2025) and Veterinary Directorate of Ministry of Agriculture, Forestry and Water Management of RS (approval number: 003447280/2025; date: 25 August 2025.) in accordance with the European Communities Council Directive (2010/63/EU) for animal experiments. Care was taken to minimize pain and discomfort of the animals, and the number of animals used was limited to the minimum required to obtain reliable and reproducible results. All experimental procedures were carried out in accordance with the UK Animals (Scientific Procedures) Act 1986 and associated guidelines and the National Institutes of Health guide for the care and use of Laboratory animals (NIH Publications No. 80-23, revised 1996). The animals were obtained from the animal facility of the VINČA Institute of Nuclear Science—National Institute of the Republic of Serbia, University of Belgrade. The animals were originally purchased from Charles River Laboratories (Wistar WU rats, strain code 619), outbred, without genetic modification and other prior procedures.

A total of 130 female Wistar rats (2.5 months old) were used in this study. Female animals were selected based on epidemiological evidence indicating a higher prevalence of BZD use among women compared to men. Previous studies have shown that women are affected by anxiety and fear-related disorders more compared to men [55], and are more frequently prescribed BZDs, exhibiting higher rates of long-term use, with reported usage approximately 1.5–2-fold greater than in men [56,57,58]. Therefore, the use of female animals enhances the translational relevance of the present study by better reflecting the clinical population most affected by BZD exposure.

### 4.2. Experimental Design and Drug Administration

Animals were housed under standard laboratory conditions (12 h light/dark cycle, controlled temperature and humidity) with ad libitum access to food and water. Rats were randomly assigned to the following experimental groups: vehicle control (VEH), 36 h of ALP withdrawal (36 h ALP), and 96 h of ALP withdrawal (96 h ALP). Prior to the start of the experiment, animals were handled daily for one week to habituate them to the experimenters and minimize stress during subsequent procedures.

**Figure 7:**
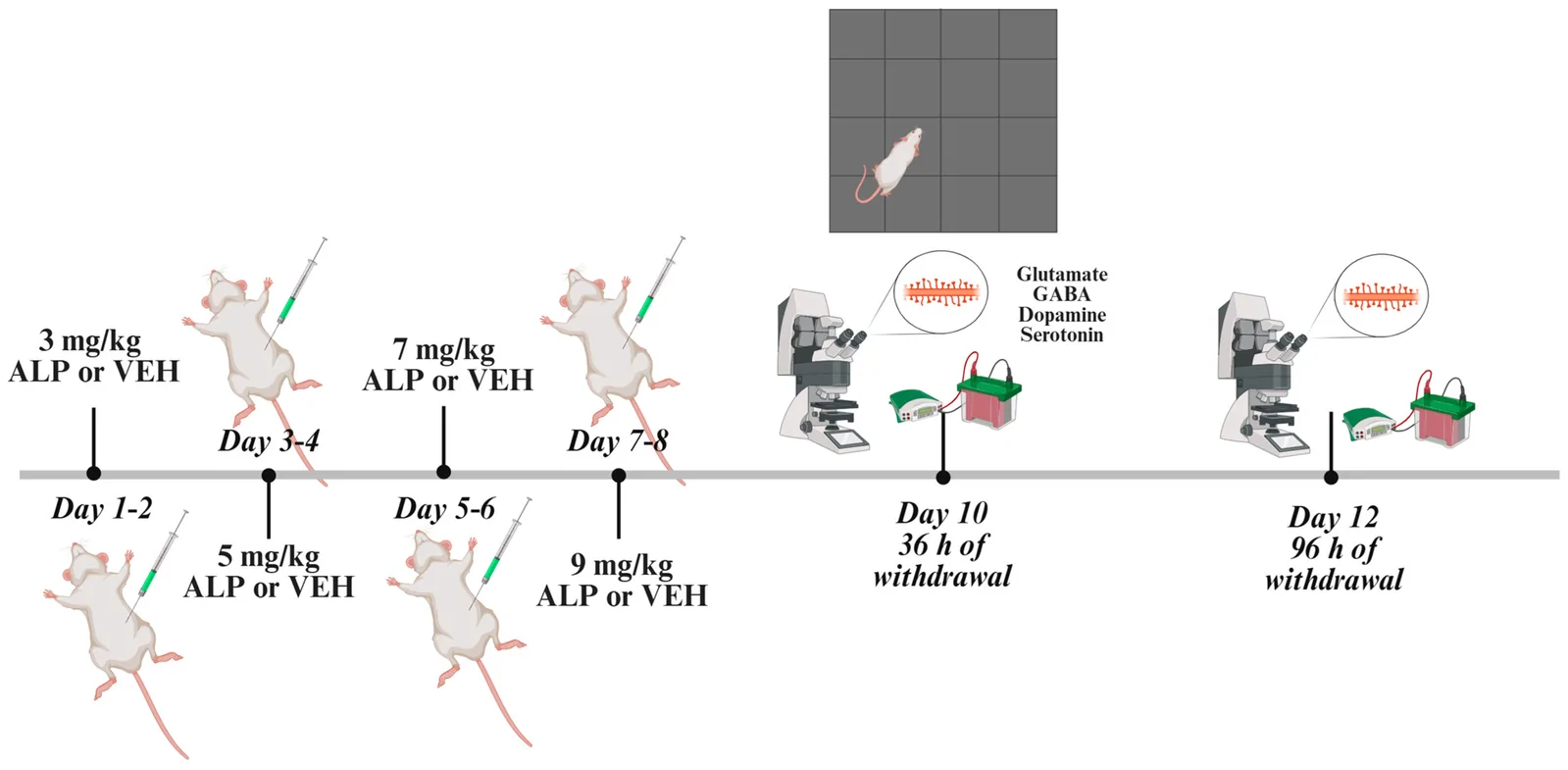
Experimental timeline. Adult female Wistar rats received an 8-day-long alprazolam (ALP) treatment in an escalating-dose regimen (3, 5, 7, and 9 mg/kg, i.p.) or vehicle (VEH), as indicated. Behavioral assessment using the Open Field Test (OFT) was performed after 36 h of withdrawal. Neurotransmitter and neuromodulator levels were determined after 36 h of withdrawal, whereas synaptic protein expression (Western blot) and dendritic spine density and morphology (Golgi–Cox staining) were analyzed after both 36 h and 96 h of withdrawal. Created in BioRender. Zaric Kontic, M. (2026) https://BioRender.com/zywx4ne (accessed on 31 August 2026).

As ALP is poorly soluble in aqueous solutions, Tween 80 was added to facilitate its dissolution and ensure uniform drug administration. Accordingly, ALP was prepared in 0.5% Tween 80 in saline. Animals in the treatment groups received ALP in escalating doses ranging from 3 mg/kg to 9 mg/kg, administered intraperitoneally. Each dose was maintained for two consecutive days, with two administrations per day (9:00 a.m. and 9:00 p.m.) to extend drug exposure across the daily treatment period and to approximate typical minimum twice-daily clinical dosing regimens of alprazolam [5,6]. Schematic presentation of the experimental timeline is depicted in Figure 7. The control animals received vehicle solution consisting of 0.5% Tween 80 dissolved in physiological saline according to the same administration schedule. To minimize discomfort and local tissue irritation, injections were alternated between the left and right sides of the abdomen between morning and evening administrations.

The applied ALP treatment regimen was designed to provide repeated, progressively increasing drug exposure and to reflect the clinically relevant pattern of dose escalation that may occur during prolonged BZD use and may be associated with therapeutic tolerance and/or misuse [59]. The administered doses were not intended to represent direct mg/kg equivalents of therapeutic ALP doses in humans, as direct body-weight–based dose comparisons across species do not adequately account for interspecies differences in pharmacokinetics and metabolism [60]. This consideration is particularly relevant for ALP, which exhibits substantially faster elimination in rats than in humans [61]. Accordingly, intensive chronic ALP exposure paradigms have previously been employed in rats to investigate neurobiological adaptations associated with repeated treatment and subsequent withdrawal [61,62]. Thus, the present escalating-dose paradigm was intended to model progressive ALP exposure followed by spontaneous withdrawal rather than to reproduce human therapeutic dosing on a direct mg/kg basis. The age of the animals was selected based on the interspecies age-correlation principles described by Sengupta et al. (2013), which relate rat age to human developmental stages [63]. Based on these considerations, 2.5-month-old rats correspond to the young adult stage, which is broadly comparable to early adulthood in humans [63], a population frequently included in studies of BZD use [56,57,58,64].

Following the final drug administration, animals underwent withdrawal periods of either 36 h or 96 h prior to the respective experimental assessments described below.

### 4.3. Neurological and Sensorimotor Screening

As part of routine neurological and sensorimotor monitoring, animals underwent brief functional screening before initiation of the treatment protocol and again at the end of the injection period, prior to behavioral testing. The screening included negative geotaxis, visual placing, acoustic startle response, and assessment of forelimb motor function and gait. For the negative geotaxis test, animals were placed facing downward on an inclined surface, and their ability to reorient and move upward was assessed. Visual placing was evaluated by slowly moving the animal toward the edge of a horizontal surface while supporting it by the tail and observing appropriate forelimb extension and placement onto the surface. Auditory responsiveness was assessed by the presence of a startle response to a sudden acoustic stimulus. Forelimb motor function and coordination were evaluated while the animal was supported by the tail, with only the forelimbs contacting the surface, and forward movement and gait were observed.

### 4.4. Open Field Test

Open Field Test (OFT) was conducted to evaluate specific ALP withdrawal-related behaviors such as general locomotor and exploratory activity, as well as levels of anxiety-like behavior. Behavioral testing was performed exclusively after 36 h of withdrawal, as this time point was selected to validate the withdrawal-associated anxiety-like phenotype, whereas subsequent experiments focused on characterizing the temporal neurochemical, molecular, and structural adaptations associated with withdrawal while minimizing animal use in accordance with the principle of reduction. All animals included in behavioral testing were behaviorally *naïve* prior to the experiments, and each animal was tested only once. To avoid potential confounding effects of behavioral testing on the highly dynamic neurochemical and synaptic parameters examined in this study, animals subjected to the OFT were excluded from all subsequent neurochemical, molecular, and morphological analyses.

The potential influence of repeated VEH administration on behavioral parameters was further evaluated in a pilot experiment. The same animals (*n* = 12) were assessed in the OFT at baseline, before initiation of the VEH administration protocol, and reassessed approximately 36 h after the final VEH injection. The two OFT assessments were separated by a two-week interval that encompassed the VEH treatment protocol. Baseline and post-VEH measurements were analyzed as paired observations.

The OFT was performed in a secluded room to ensure both acoustic and visual isolation. Prior to testing, animals were allowed a habituation period of at least 60 min to the behavioral testing environment to minimize stress-related variability. All animal handling related to behavioral assessments was conducted by a single researcher to ensure consistency in procedures. Control and treated animals were tested in parallel on the same day to maintain uniform experimental conditions. To eliminate olfactory cues, the apparatus was thoroughly cleaned between sessions using 70% ethanol and water and dried by wiping with dry paper to remove residual odor and liquid.

The OFT was performed in a relatively large cohort of animals (VEH: *n* = 32; 36 h ALP: *n* = 35), as it served as the primary behavioral assay for validating the withdrawal-associated phenotype. At the beginning of the test, animals were placed in the center of a black arena (100 × 100 × 50 cm), and their activity was recorded for 10 min and analyzed in real time using ANY-maze software (version 7.65, 64-bit; Stoelting Co., Wood Dale, IL, USA).

The arena was virtually divided into 16 equal squares, with the central four squares defined as the central zone, which was more brightly illuminated (120 lux) compared to the peripheral (10 lux) and corner areas (10 lux). Behavioral parameters included time spent in the central versus peripheral zones, locomotor activity, and exploratory patterns. The center-point tracking option was used for automated detection of zone occupancy and locomotor activity.

Behavioral events were quantified using predefined detection thresholds implemented in the ANY-maze software. A central zone entry was recorded when the tracked center point remained within the central area for ≥1 s. Freezing was defined as the absence of movement for ≥10 s, while immobility episodes were defined as the absence of movement for ≥30 s.

### 4.5. Animal Euthanasia and Sample Collection

At the designated experimental endpoints (36 h and 96 h withdrawal), animals were euthanized by rapid decapitation using a guillotine for small laboratory animals (Harvard Apparatus, Holliston, MA, USA). Anesthetic agents were not used prior to euthanasia in order to avoid potential alterations in neurochemical parameters.

Immediately following decapitation, brains were rapidly removed from the skull and processed for further analyses. Depending on the downstream experimental procedure, whole brains or amygdaloid regions were isolated as quickly as possible to minimize post-mortem changes and preserve biochemical integrity. The amygdaloid region was isolated by dissecting the dorsolateral portion of the cortico-subcortical tissue corresponding to the amygdala, approximately spanning anteroposterior coordinates −1.80 to −3.24 mm according to the Paxinos and Watson rat brain atlas [54].

All procedures were carried out in accordance with ethical guidelines, with efforts made to minimize animal suffering and distress.

### 4.6. Golgi-Cox Staining

Structural neuronal changes in the basolateral amygdala (BLA) were assessed using Golgi-Cox impregnation. The BLA was selected for its role as a major input nucleus, receiving extensive cortical and subcortical projections and participating in emotional learning, fear processing, stress responsiveness, and anxiety regulation. Through reciprocal interactions with the prefrontal cortex, hippocampus, hypothalamus, and central amygdala, the BLA plays a critical role in coordinating adaptive responses to emotionally salient stimuli and maintaining emotional homeostasis [16,19]. Modified Golgi–Cox staining for visualization of dendritic morphology and spine density was performed with slight modifications according to previously established protocols [53,65,66,67]. In brief, brains (*n* = 4 animals per group) were rapidly removed from the skull, cut into two halves along the midsagittal plane, and immersed in an impregnating solution consisting of potassium chromate, potassium dichromate, and mercury (II) chloride. Tissue was stored in the dark for 15 days. Following impregnation, brains were washed in distilled water to remove excess solution and subsequently cryoprotected in 30% sucrose solution prepared in 0.2 M phosphate buffer for 14 days. Coronal sections (100 μm thick) were obtained using a cryostat and mounted onto gelatin-coated slides. Sections were air-dried for 1–2 h and stored in the dark at room temperature until further processing. For development, sections were incubated in deionized water for 5 min and 50% ethanol solution for another 5 min, followed by 1% lithium carbonate (Li_2_CO_3_) solution for 25 min and washing in deionized water for 5 min. Sections were then dehydrated through a graded ethanol series (70–100%) and cleared in xylene for 20 min. No fluorescent probe was added to the Golgi–Cox-impregnated sections. Finally, sections were coverslipped using DPX mounting medium. Dendritic spine analysis was performed on pyramidal neurons within the BLA using a confocal laser-scanning microscope (LSM 510, Carl Zeiss GmbH, Jena, Germany) and a HeNe (633 nm) laser with a long-pass 650 nm filter. The BLA was identified according to the Paxinos and Watson rat brain atlas [54], and neuronal sampling was restricted to coronal sections spanning approximately −1.80 to −2.52 mm relative to Bregma to ensure anatomically comparable sampling across experimental groups. Neurons were selected based on clear staining, minimal overlap with neighboring cells, and intact dendritic architecture. Z-stack images of Golgi-Cox-stained secondary and tertiary apical dendritic branches (1024 × 1024 pixels, 1 pixel = 0.09 μm, up to 20 μm total on the *Z*-axis, optical section thickness = 0.5 μm, i.e., up to 40 images per stack) were captured using a 100 × (DIC, N*_A_* = 1.4) oil objective and monochrome AxioCam ICm1 camera (Carl Zeiss GmbH, Germany). Detailed information on optical signal validation and image acquisition and processing is provided in the Supplementary Material (Section Supplementary Methods). For each animal, at least three neurons were analyzed, with a minimum of five dendritic branches per neuron. Spine density and morphology were quantified on dendritic segments of 15 μm in length using ImageJ software, version 1.54g (NIH, Bethesda, MD, USA) in combination with Reconstruct software, version 1.1.0.0 (2007) [68]. Spines were classified into six morphological categories: thin, long-thin, stubby, branched, mushroom, and filopodia. Classification was based on quantitative morphological criteria, including spine length, head diameter, and branching characteristics, with predefined threshold values applied consistently across all samples as described in [65]. Care was taken to track segment origin, ensuring representation across neurons and animals within each group. Quantification was performed in a blinded manner, with the experimenter unaware of group allocation during analysis.

### 4.7. Synaptosomal Preparation and Western Blot Analysis

Synaptosomal fractions were isolated using the Syn-PER™ Synaptic Protein Extraction Reagent (Cat. No. 87793, Thermo Fisher Scientific, Waltham, MA, USA), according to the manufacturer’s instructions.

Protein concentration was determined using the Pierce™ BCA Protein Assay Kit (Thermo Fisher Scientific, USA), following the manufacturer’s protocol.

For Western blot analysis (*n* = 5–7 per group), protein samples (16 μg per lane) were subjected to sodium dodecyl sulfate–polyacrylamide gel electrophoresis using a 10% resolving gel in a Bio-Rad tetra electrophoresis system. Proteins were then transferred onto polyvinylidene difluoride membranes (Cat. No. IPVH00010, Immobilon-P, Millipore, Burlington, MA, USA) using a Bio-Rad Trans-Blot Turbo transfer system. A pre-stained protein ladder (Cat. No. 26616, PageRuler™, Thermo Fisher Scientific, Waltham, MA, USA) was used as a molecular weight marker. Membranes were blocked in 5% non-fat dry milk (Cat. No. M7409, Sigma-Aldrich, St. Louis, MO, USA) and subsequently incubated with primary antibodies (Table 1), followed by appropriate secondary antibodies (Table 2). Protein bands were visualized using a chemiluminescent substrate based on luminol (Immobilon Western Chemiluminescent HRP Substrate, Millipore, Burlington, MA, USA) and detected using an imaging system (iBright FL1500, Thermo Fisher Scientific, Waltham, MA, USA). Densitometric analysis was performed using ImageJ software (NIH, Bethesda, MD, USA). Protein expression levels were normalized to β-actin as a loading control and expressed as a percentage of the mean ± SEM relative to β-actin levels.

Full-length uncropped Western blot images corresponding to Figure 2, Figure 3, Figure 4 and Figure 5 are provided in the Supplementary Material.

### 4.8. Examination of Neurotransmitter and Neuromodulator Levels

Neurotransmitter and neuromodulator levels were determined in the amygdaloid region to assess neurochemical alterations associated with ALP withdrawal. Neurochemical changes were assessed only after 36 h of withdrawal, as this time point coincided with the behavioral assessment and therefore allowed examination of neurochemical alterations at the same withdrawal stage at which the behavioral phenotype was characterized. Additional neurochemical analyses at later withdrawal stages were not performed to limit animal use in accordance with the principle of reduction.

#### 4.8.1. Glutamate Assay

Glutamate levels were determined using a commercially available assay kit (Cat. No. ab83389, Abcam, Cambridge, UK, *n* = 8 per group), following the manufacturer’s protocol, with an additional deproteinization and neutralization step. Briefly, 1 M perchloric acid (PCA) was added to the sample, followed by vortexing. Samples were then centrifuged at 13,000 rpm for 2 min, and the supernatant was collected. Neutralization was performed by adding 2 M KOH (34 μL per 100 μL of supernatant), followed by vortexing. Tubes were carefully opened due to gas formation during the reaction. The pH of the samples was measured and adjusted to a range of 6.5–8.0 using either 0.1 M or 2 M KOH, or 1 M PCA as needed. The total volume of added PCA and KOH during the neutralization step was taken into account in the final calculations. Samples were then centrifuged at 13,000 rpm for 15 min, and the resulting supernatant was used for further analysis according to the kit protocol. Glutamate content was expressed as μg/mL of glutamate per mg of sample tissue.

#### 4.8.2. GABA Assay

GABA levels were measured using a commercially available assay kit (Cat. No. E-BC-K852-M, Elabscience, Wuhan, China), according to the manufacturer’s instructions. Tissue samples (VEH group: *n* = 7 per group, 36 h ALP group: *n* = 9) were prepared and processed following the provided protocol, and absorbance was measured using a microplate reader. GABA concentrations were calculated based on the standard calibration curve and expressed relative to tissue content. Neurotransmitter concentrations were expressed as μmol/g tissue.

#### 4.8.3. Dopamine and Serotonin Analysis

Dopamine and serotonin levels were quantified using high-performance liquid chromatography (HPLC) with electrochemical detection. Brains were rapidly removed, and the amygdala was dissected for analysis. Tissue samples (*n* = 7 per group) were homogenized in deproteinization solution (1 mg tissue/10 μL), followed by sonication (3 × 10 s) and centrifugation (10 min, 13,500 rpm). The resulting supernatants were collected for analysis. An aliquot of each sample (40 μL) was injected into an UltiMate 3000 HPLC system (Thermo Fisher Scientific, USA) equipped with a C18 column. The mobile phase consisted of 100 mM ammonium formate buffer (pH 3.6) and methanol, with an initial ratio of 98:2 (*v*/*v*), delivered at a flow rate of 500 μL/min. Under these conditions, dopamine and serotonin were separated and detected using an electrochemical detector set at 850 mV and 25 °C. Data was acquired and analyzed using Chromeleon 7 Chromatography Data System (Thermo Fisher Scientific, USA). Neurotransmitter/neuromodulator concentrations were expressed as pmol/mg tissue.

### 4.9. Statistical Analysis

The required number of animals for the behavioral and molecular experiments was determined using G*Power software (version 3.1), with expected effect sizes based on our previous chronic ALP experiment [10]. For the behavioral analysis, an a priori power analysis assuming Cohen’s d = 0.80, α = 0.05, and power (1 − β) = 0.90 was used, whereas for the molecular analyses Cohen’s f = 1.0, α = 0.05, and power (1 − β) = 0.95 were applied. Detailed effect size derivations, statistical test specifications, and G*Power sample size calculations are provided in the Supplementary Material (Figures S3 and S4). The Golgi–Cox analysis (*n* = 4 animals/group) was considered exploratory and was not included in the formal power-based sample size determination. All statistical analyses were performed using GraphPad Prism 9.0 software (GraphPad Software, San Diego, CA, USA). Prior to analysis, data were tested for normality to confirm the suitability of parametric tests. Behavioral comparisons between the independent VEH and 36 h ALP withdrawal groups were performed using an unpaired Student’s *t*-test. For the pilot analysis evaluating the potential effect of repeated VEH administration on OFT behavior, baseline and post-VEH measurements obtained from the same animals were analyzed using a paired Student’s *t*-test. All behavioral data were presented as mean ± standard deviation (SD). Data obtained from biochemical assays and HPLC measurements were also analyzed using an unpaired Student’s *t*-test. Golgi–Cox morphometric data and Western blot results were analyzed using one-way analysis of variance (ANOVA), followed by Tukey’s post hoc test for multiple comparisons. Protein expression levels in Western blot analyses were normalized to β-actin as a loading control and expressed as a percentage of the mean value of vehicle (VEH) group. All these results are presented as the mean ± standard error of the mean (SEM). Differences were considered statistically significant at *p* < 0.05.

## 5. Conclusions

Collectively, the present findings support a model in which ALP withdrawal induces coordinated adaptations across multiple levels of biological organization, spanning neurotransmitter systems, synaptic proteins, dendritic spine architecture, and behavior. Rather than reflecting isolated alterations within individual neurotransmitter systems, the observed findings indicate integrated remodeling of excitatory and inhibitory synaptic compartments within the amygdala. Enhanced glutamatergic signaling, heterogeneous adaptations of GABAergic synapses, altered dopaminergic modulation, and selective remodeling of dendritic spine populations together suggest that ALP withdrawal is associated with coordinated reorganization of amygdala synaptic networks, which may contribute to increased withdrawal-associated anxiety-like behavior.

To the best of our knowledge, this study is among the first to integrate comprehensive behavioral characterization with neurochemical analyses, synaptic protein profiling, and dendritic spine morphology within the same experimental model of ALP withdrawal. Notably, this integrative approach was applied in adult female rats, a population that remains substantially underrepresented in preclinical models of BZD withdrawal despite the higher prevalence of anxiety disorders and more frequent clinical use of BZDs in women. By linking neurotransmitter alterations with molecular and structural remodeling of excitatory and inhibitory synapses, our findings provide a broader framework for understanding the neurobiological mechanisms underlying BZD withdrawal. These findings identify coordinated synaptic plasticity within the amygdala as a potential substrate for withdrawal-associated anxiety and provide a foundation for future studies aimed at improving the understanding and clinical management of BZD withdrawal.

## Figures and Tables

**Table 1:** List of primary antibodies used in Western blot analysis.

Primary Antibodies			
Antibody	Manufacturer, Cat. No., RRID	Host	Dilution
α1-GABAAR	Sigma Aldrich, G4416; AB_477016	Rabbit	1:2000
vGAT	Millipore, # AB2257; AB_1587623	Rabbit	1:2500
GAD65	Sigma-Aldrich, # MAB5406, AB_2278725	Mouse	1:2000
GluR1	Cell Signaling Technology, #3838; AB_2732897	Rabbit	1:1000
NR1	Cell Signaling Technology, #5704; AB_1904067	Rabbit	1:3000
NR2A	Merck Millipore, #07-632; AB_310837	Rabbit	1:3000
NR2B	Abcam, 93610; AB_10561972	Mouse	1:2500
vGluT1	Abcam, 134283; AB_2923539	Mouse	1:1500
EAAT1	Cell Signaling Technology, #5684T, AB_10671594	Rabbit	1:1000
EAAT2	Cell Signaling Technology, #3838; AB_2190743	Rabbit	1:1000
D1R	Abcam, ab81296; AB_2814742	Rabbit	1:500
D2R	FineTest, FNab02533; AB_3751482	Rabbit	1:500
SYN	Santa Cruz Biotechnology, # sc-9116, RRID; AB_2199007	Rabbit	1:20,000
PSD-95	Millipore, MAB1598; AB_11212185	Mouse	1:2500
Gephyrin	Cell Signaling Technology, #14304; AB_2798443	Rabbit	1:1000
β—actin	Abcam, ab49900; AB_867494	Mouse	1:5000

**Table 2:** List of secondary antibodies used in Western blot analysis.

Secondary Antibodies		
Antibody	Manufacturer, Cat No; RRID	Dilution
Anti-Mouse IgG HRP	R&D systems, Bio-techne, HAF007; AB_357234	1:10,000
Anti-Rabbit IgG	Thermo Fisher Scientific Invitrogen, # 31460; AB_228341	1:10,000

## Data Availability

The original contributions presented in the study are included in the article/Supplementary Material, further inquiries can be directed to the corresponding author.

## Supplementary Materials

The following supporting information can be downloaded at https://www.mdpi.com/article/10.3390/ph19091433/s1, Figure S1: Open field test (OFT) behavioral measures at baseline and following repeated vehicle administration; Figure S2: Additional locomotion-adjusted and compartment-specific behavioral measures obtained in the open field test (OFT); Figure S3: G*Power sample-size calculation for the behavioral experiment in the present study; Figure S4: G*Power sample-size calculation for the molecular analyses in the present study. Reference [69] was cited in supplementary materials.

## Abbreviations

The following abbreviations are used in this manuscript:

ALP	Alprazolam
AMPAR	α-amino-3-hydroxy-5-methyl-4-isoxazolepropionic acid receptor
ANOVA	Analysis of variance
BLA	Basolateral amygdala
BZD	Benzodiazepine
D1R	Dopamine D1 Receptor
D2R	Dopamine D2 Receptor
EAAT1	Excitatory amino acid transporter 1
EAAT2	Excitatory amino acid transporter 2
GABA	γ-Aminobutyric acid
GABA_A_R	γ-Aminobutyric acid type A receptor
GAD65	Glutamic acid decarboxylase 65
GC	Goldgi-Cox staining
HPLC	High-performance liquid chromatography
NMDAR	N-methyl-D-aspartate receptor
OFT	Open field test
PCA	Perchloric acid
PSD-95	Postsynaptic density protein 95
SD	Standard deviation
SEM	Standard error of the mean
SYN	Synaptophysin
vGAT	Vesicular GABA amino acid transporter
vGluT1	Vesicular glutamate transporter 1
