# Supplementary material for: Synaptic Adaptations in the Amygdala During Alprazolam Withdrawal: Insights from a Female Rat Model of Benzodiazepine Withdrawal
Source: Pharmaceuticals (Basel). 2026 Sep 10;19(9):1433. doi: 10.3390/ph19091433 (PMC13610263; doi:10.3390/ph19091433)
Supplement: Supplementary file 1 [file pharmaceuticals-19-01433-s001.zip › pharmaceuticals-4449103-supplementary.pdf]

Supplementary Material

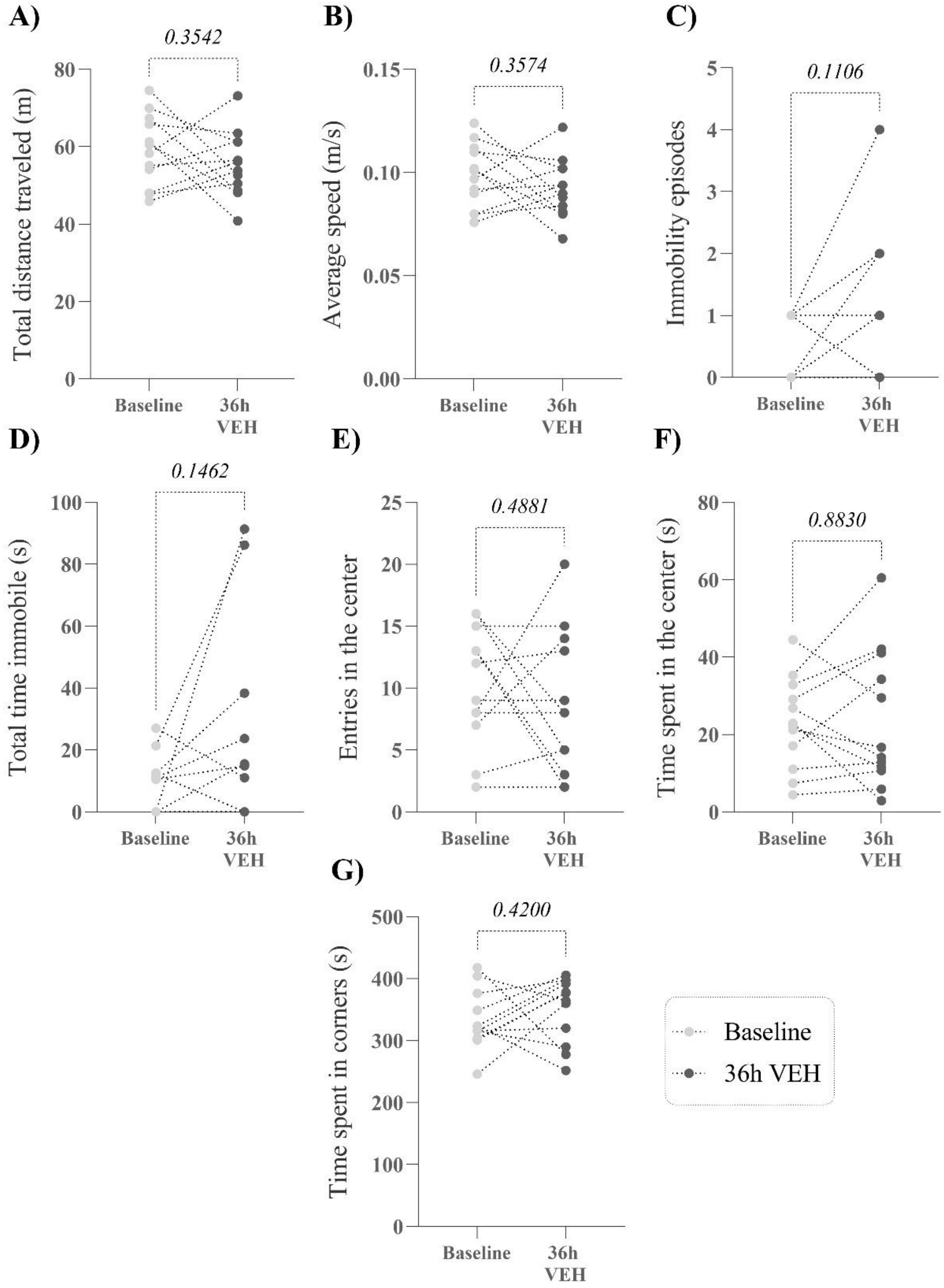

Figure S1. Open field test (OFT) behavioral measures at baseline and following repeated vehicle administration. Baseline OFT assessment was performed before initiation of the vehicle-treatment protocol. *Following a two-week interval, the same animals were reassessed in the OFT approximately 36 h after the final VEH injection. (A) total distance traveled, (B) average speed, (C) number of immobility episodes, (D) total time immobile, (E) number of entries into the center, (F) time spent in the center, and (G) time spent in the corners of the arena. Connected dots represent paired measurements obtained from the same individual animals*

at baseline and 36 h after the final VEH injection. No significant differences were observed between baseline and post-VEH measurements for any of the analyzed behavioral parameters. Precise numerical p *values are shown above the respective comparisons.*

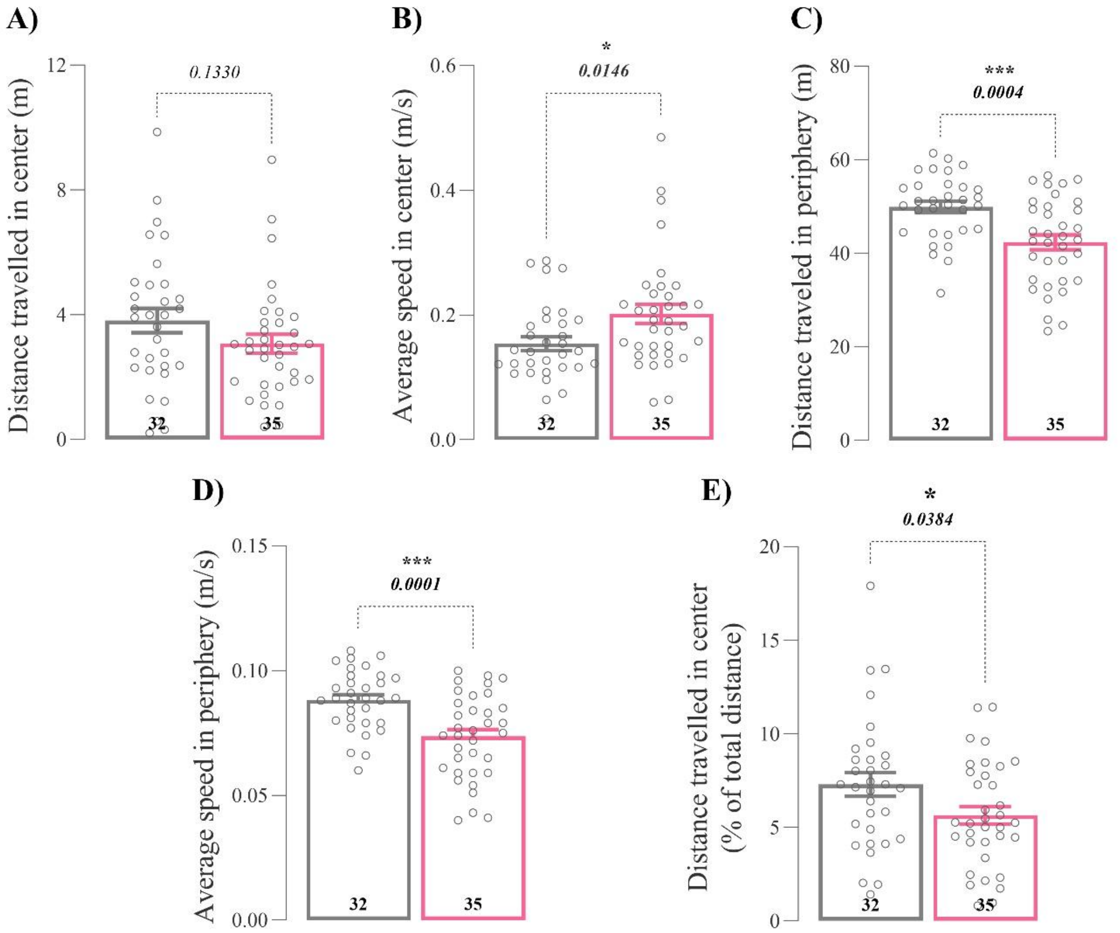

Figure S2. Additional locomotion-adjusted and compartment-specific behavioral measures obtained in the open field test (OFT). *The test was performed approximately 36 h after the last ALP injection. (A) distance travelled in the center, (B) average speed in the center, (C) distance travelled in the periphery, (D) average speed in the periphery, and (E) distance travelled in the center expressed as a percentage of the total distance travelled by each animal. The locomotion-adjusted measure shown in panel E was calculated as distance travelled in the center/total distance travelled × 100. Dots in the graphs represent the values of individual animals. Numbers at the bottom of the graphs indicate the number of animals included in the analysis. Data are expressed as mean ± SD. Significance is shown in the graphs as precise numerical* p *values and star symbols indicating significant differences between the VEH and 36 h ALP groups: * $p < 0.05$, *** $p < 0.001$.*

Full-length uncropped Western blot images

The images presented below are full-length, uncropped Western blot images corresponding to the protein-expression analyses shown in Figures 2–5. For visualization purposes, the original images acquired using the iBright Imaging System were inverted to facilitate inspection of the protein bands. No cropping, brightness or contrast adjustment, or other image processing was applied. Each protein-of-interest blot is presented together with its corresponding β-actin loading control. Completely unprocessed original iBright image files are provided separately with the submission.

Western blot data corresponding to Figure 2

vGLUT1

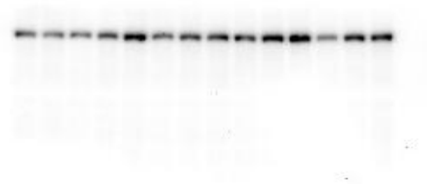

Corresponding β-actin

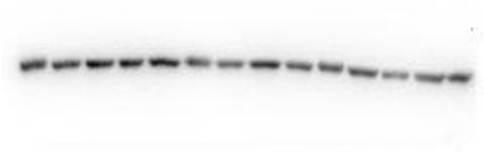

GluR1

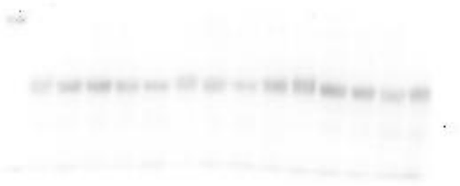

Corresponding β-actin

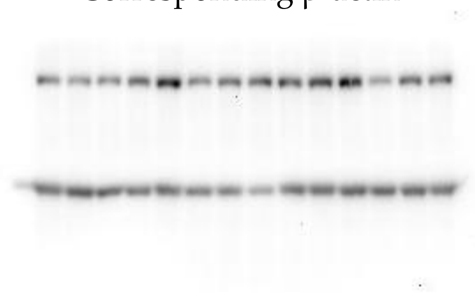

NR1

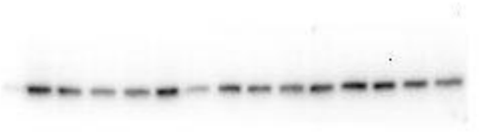

Corresponding β-actin

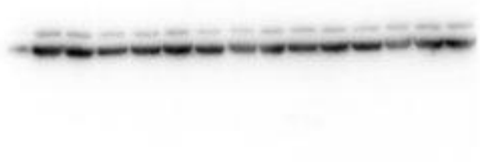

NR2A

Corresponding β-actin

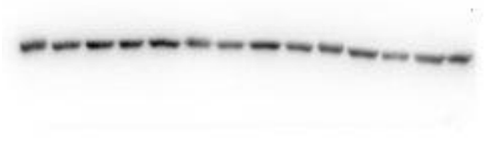

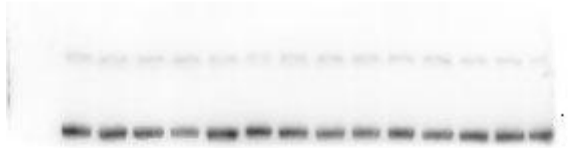

NR2B

Corresponding β-actin

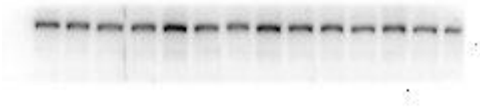

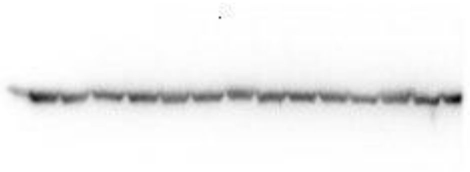

PSD-95

Corresponding β-actin

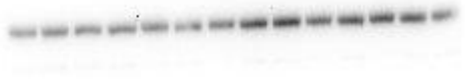

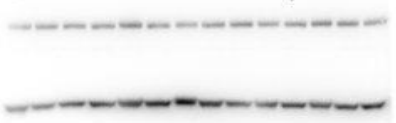

EAAT1

Corresponding β-actin

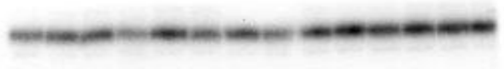

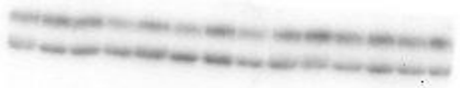

EAAT2

Corresponding β-actin

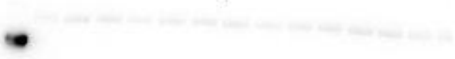

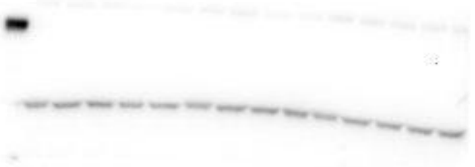

Western blot data corresponding to Figure 3

vGAT

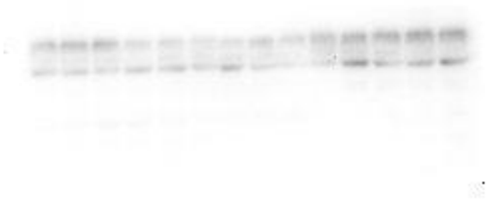

Corresponding β-actin

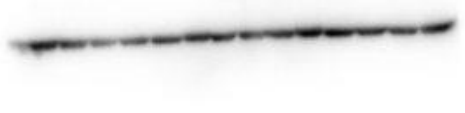

GAD65

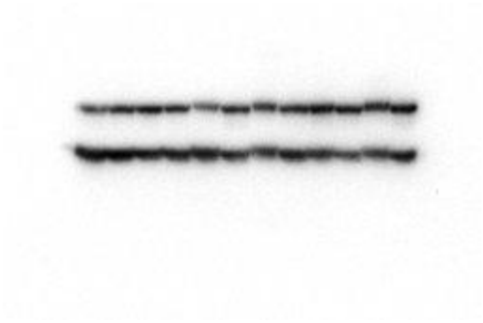

Corresponding β-actin

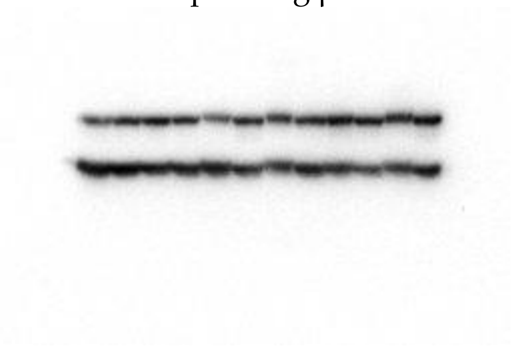

Gephyrin

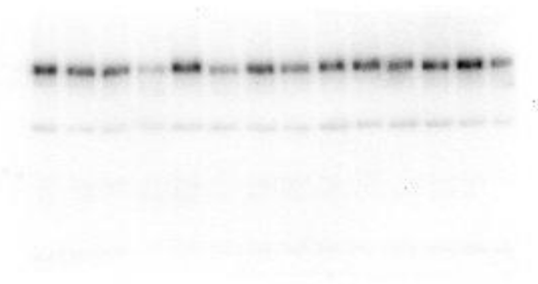

Corresponding β-actin

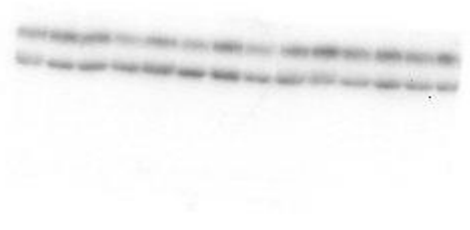

α1 GABAAR

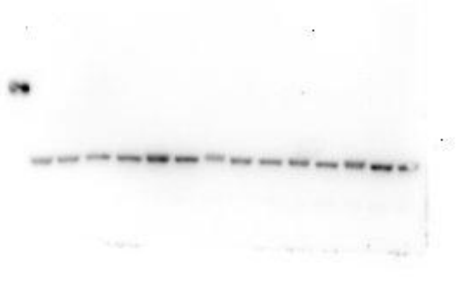

Corresponding β-actin

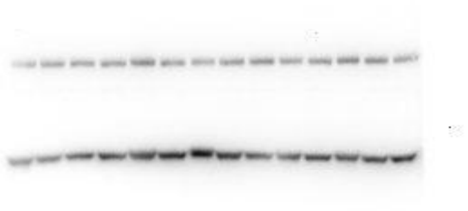

Western blot data corresponding to Figure 4

D1R

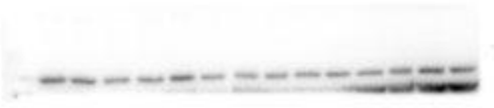

Corresponding β-actin

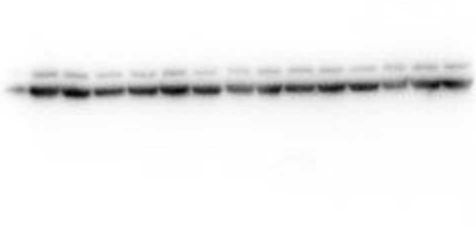

D2R

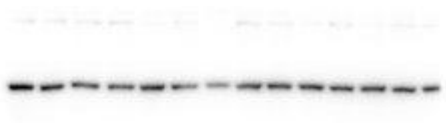

Corresponding β-actin

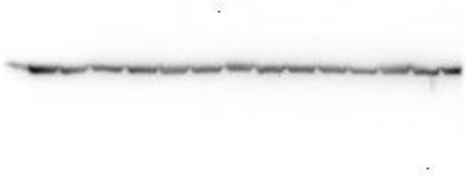

Western blot data corresponding to Figure 5

SYN

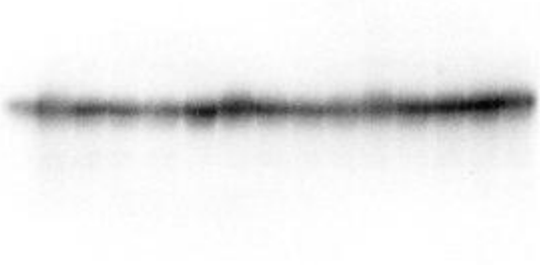

Corresponding β-actin

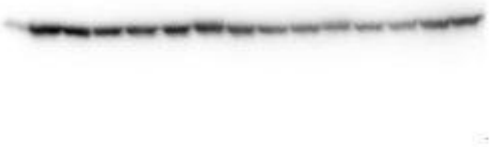

Supplementary information on sample-size rationale and power analysis

To provide additional transparency regarding the sample-size rationale for the present study, expected effect magnitudes for the behavioral and molecular experiments were informed by data obtained in our previous chronic alprazolam (ALP) experiment. Because the behavioral and molecular datasets in the present study involve different statistical designs, the corresponding effect-size metrics and power calculations are presented separately below.

The previous experimental data are used here only to support the selection of the expected effect sizes. The G*Power calculations shown below refer to the sample-size requirements for the present study.

S1. Behavioral analysis

The behavioral experiment in the present study involved comparison of two independent groups (VEH and 36 h ALP withdrawal). Accordingly, Cohen's d was used as the standardized effect-size metric.

The expected behavioral effect magnitude was informed by open field test data obtained in our previous chronic ALP experiment. For the representative center-entry endpoint, comparison of VEH and ALP-treated animals yielded $t(24) = 2.103$, with $n = 13$ animals per group. For two independent groups of equal size, Cohen's d can be derived from the t statistic as:

$$d = t \times \sqrt{(1/n_1 + 1/n_2)}$$

$$d = 2.103 \times \sqrt{(1/13 + 1/13)}$$

$$d = 2.103 \times 0.3922 = 0.825$$

Thus, the previously observed behavioral effect corresponded to Cohen's $d \approx 0.83$. An expected effect size of Cohen's $d = 0.80$ was therefore used for the sample-size calculation for the behavioral experiment in the present study.

G*Power calculation for the present behavioral experiment

The following parameters were entered in G*Power:

Test family: t tests
Statistical test: Means: difference between two independent means (two groups)
Type of power analysis: A priori – compute required sample size
Tail(s): Two

Effect size: Cohen's d = 0.80
α error probability: 0.05
Power (1–β): 0.90
Allocation ratio N2/N1: 1

Under these assumptions, the calculation indicated a required sample size of approximately **34 animals per group (approximately 68 animals in total)**.

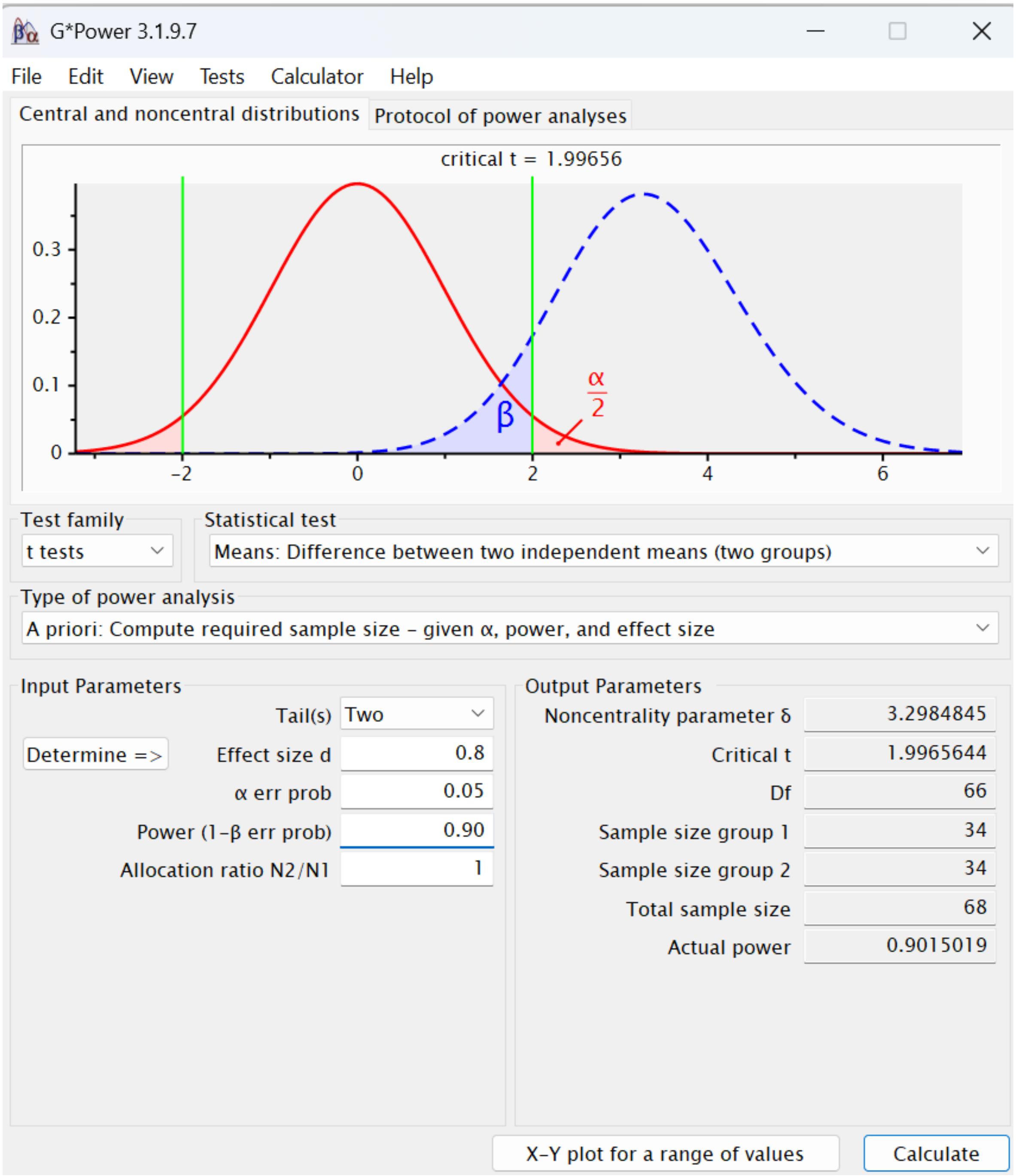

*Supplementary Figure S3. G*Power sample-size calculation for the behavioral experiment in the present study.*

The calculation was performed for a two-tailed independent-samples t-test using an expected effect size of Cohen's d = 0.80, informed by the magnitude of the

behavioral effect observed in our previous chronic ALP experiment, with $\alpha = 0.05$, power $(1-\beta) = 0.90$, and equal allocation between groups.

S2. Molecular analyses

The molecular analyses in the present study involved three experimental groups (VEH, 36 h ALP, and 96 h ALP) evaluated by one-way ANOVA. Cohen's f was therefore used as the standardized effect-size metric for the corresponding sample-size calculation.

The expected magnitude of molecular effects was informed by representative protein-expression outcomes obtained in our previous chronic ALP experiment. In that experiment, the proportion of variance explained by the group factor was $R^2 = 0.4647$ for the GABA receptor analysis and $R^2 = 0.5118$ for the NR2B analysis. For an ordinary one-way ANOVA, these values were used as η^2 and converted to Cohen's f according to:

$$f = \sqrt{[\eta^2/(1-\eta^2)]}$$

For the GABA receptor analysis:

$$f = \sqrt{[0.4647/(1-0.4647)]} = \sqrt{(0.4647/0.5353)} = 0.932$$

For the NR2B analysis:

$$f = \sqrt{[0.5118/(1-0.5118)]} = \sqrt{(0.5118/0.4882)} = 1.024$$

Thus, representative molecular outcomes from the previous chronic ALP experiment yielded Cohen's f values of approximately 0.93–1.02. Based on this empirically observed effect range, Cohen's f = 1.0 was selected as the representative expected effect magnitude for the molecular sample-size calculation in the present study.

G*Power calculation for the present molecular analyses

The following parameters were entered in G*Power:

Test family: F tests
Statistical test: ANOVA: fixed effects, omnibus, one-way
Type of power analysis: A priori – compute required sample size
Effect size: Cohen's f = 1.0
α error probability: 0.05
Power (1−β): 0.95
Number of groups: 3

The calculation indicated a required total sample size of **21 animals (n = 7 per group)**, with numerator df = 2, denominator df = 18, critical F ≈ 3.555, and actual power ≈ 0.972.

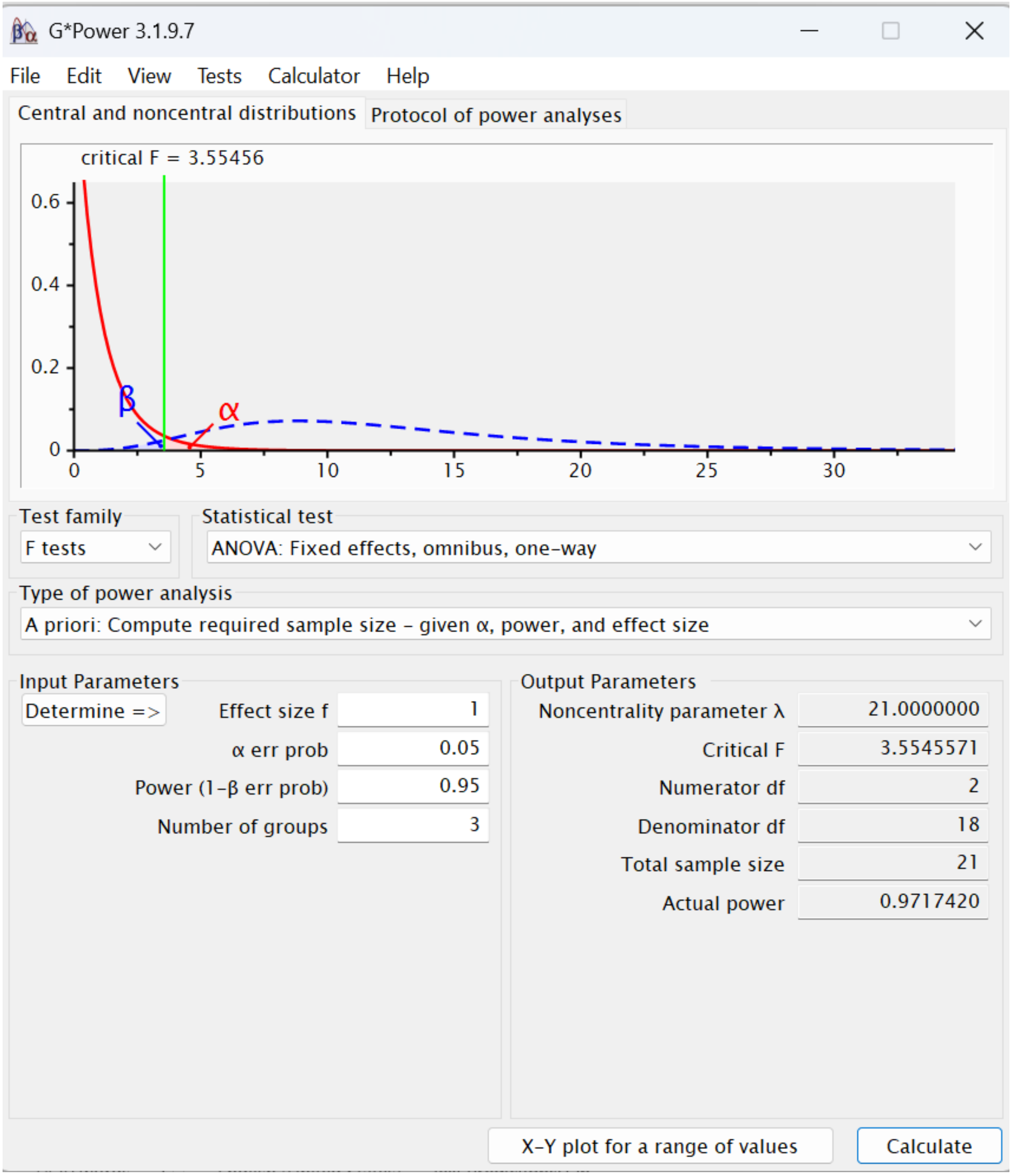

*Supplementary Figure S4. G*Power sample-size calculation for the molecular analyses in the present study.*

The calculation was performed using a fixed-effects omnibus one-way ANOVA with three experimental groups. Based on effect magnitudes observed for representative molecular endpoints in our previous chronic ALP experiment, Cohen's f = 1.0 was used as the expected effect size, with $\alpha = 0.05$ and power $(1-\beta) = 0.95$. The calculation indicated a required total sample size of 21 animals (n = 7 per group; actual power ≈ 0.972).

S3. Considerations regarding the Golgi–Cox morphological analysis

The Golgi–Cox morphological component requires separate consideration from the behavioral and molecular experiments. Morphological measurements were obtained from four animals per experimental group, with multiple neurons and dendritic segments sampled from each animal. The individual animal therefore represents the independent experimental unit, whereas neurons and dendritic segments represent subsamples nested within animals.

Following re-evaluation of the statistical approach, dendritic spine outcomes were analyzed at the animal level, thereby avoiding treatment of multiple dendritic segments obtained from the same animal as independent biological replicates.

Given the limited number of independent animals (n = 4 per group), the Golgi–Cox component is not presented as being adequately powered to detect small or moderate animal-level effects. These findings are therefore regarded as preliminary/exploratory structural observations and are interpreted cautiously. The limited animal-level sample size has also been explicitly acknowledged as a limitation of the study.

The number of neurons or dendritic segments examined increases the extent of morphological sampling within each animal but does not increase the number of independent experimental units.

Summary of the sample-size rationale

ANALYSIS	STATISTICAL DESIGN	PRIOR EFFECT ESTIMATE	EFFECT SIZE USED	A	POWER	CALCULATED SAMPLE SIZE
**BEHAVIORAL**	Two-tailed independent-samples t-test	d = 0.825	d = 0.80	0.05	0.90	≈34/group
**MOLECULAR**	One-way ANOVA, 3 groups	f = 0.932–1.024	f = 1.00	0.05	0.95	21 total; 7/group
**GOLGI–COX**	Animal-level, 3 groups	—	—	—	—	4/group; exploratory

Supplementary Methods: Optical validation and confocal imaging of Golgi–Cox-impregnated neurons

During optimization, Golgi–Cox-impregnated sections were excited using the 633-nm HeNe laser of a Zeiss LSM 510 confocal microscope. A strong signal delineating neuronal somata, dendritic branches and individual dendritic spines was detected using both a 585–650-nm band-pass filter and a 650-nm long-pass filter, with signal being consistently stronger with LP than with BP filter. Because the 585–650-nm filter includes the 633-nm excitation line, whereas the long-pass filter nominally collects wavelengths above the excitation wavelength, this finding argues against the detected signal being produced exclusively by elastic reflection of the excitation laser. Instead, it supports the presence of a red-shifted optical emission associated with the Golgi–Cox deposits. In contrast, no corresponding signal was detected in the shorter-wavelength detection channels. Importantly, imaging of the same microscopic field using transmitted-light and confocal microscopy demonstrated complete and identical spatial correspondence between the conventionally visible Golgi–Cox deposits and the structures detected in the confocal channel. The signal detected through the 585–650-nm band-pass filter may contain a contribution from light reflected or backscattered by the mercury-containing chromate deposits because this detection range includes the 633-nm excitation wavelength. However, the signal was also clearly detected through the 650-nm long-pass filter, which nominally separates the detected signal from the excitation wavelength, although only for 17 nm. This observation supports the presence of a red-shifted, laser-induced optical component associated with the Golgi–Cox deposits. Nevertheless, because the excitation wavelength lies close to the cutoff of the long-pass filter, a contribution from near-edge transmission of strongly reflected light cannot be completely excluded.

Previous studies provide evidence that Golgi–Cox deposits possess photo excitable optical properties. Kassem et al. (2018) demonstrated fluorescence of Golgi–Cox-stained tissue under multiphoton excitation [69], while Shan et al. (2022) characterized the excitation dependence of this signal using two-photon microscopy [66]. Our observations extend these findings by showing that conventional, lithium-carbonate-developed Golgi–Cox sections generate a strong stain-associated optical signal during 633-nm confocal excitation.

References (numbered according to the main manuscript):

66. Shan, Q.H.; Qin, X.Y.; Zhou, J.N. Expansion-Based Clearing of Golgi-Cox-Stained Tissue for Multi-Scale Imaging. Int. J. Mol. Sci. **2022**, 23, 3575. https://doi.org/10.3390/ijms23073575.

69. Kassem, M.S.; Fok, S.Y.Y.; Smith, K.L.; Kuligowski, M.; Balleine, B.W. A novel, modernized Golgi-Cox stain optimized for CLARITY cleared tissue. J. Neurosci. Methods **2018**, 294, 102–110. https://doi.org/10.1016/j.jneumeth.2017.11.010.
